# Leaf temperatures mediate alpine plant communities’ response to a simulated extended summer

**DOI:** 10.1002/ece3.4816

**Published:** 2018-12-28

**Authors:** Katherine F. Wentz, Jason C. Neff, Katharine N. Suding

**Affiliations:** ^1^ Remote Sensing Systems Santa Rosa California; ^2^ Environmental Studies Department University of Colorado Boulder Colorado; ^3^ Institute of Arctic & Alpine Research, Ecology & Evolutionary Biology Department University of Colorado Boulder Colorado

**Keywords:** alpine tundra, dry and wet meadows, climate change, limitations, photosynthesis model

## Abstract

We use a quantitative model of photosynthesis to explore leaf‐level limitations to plant growth in an alpine tundra ecosystem that is expected to have longer, warmer, and drier growing seasons. The model is parameterized with abiotic and leaf trait data that is characteristic of two dominant plant communities in the alpine tundra and specifically at the Niwot Ridge Long Term Ecological Research Site: the dry and wet meadows. Model results produce realistic estimates of photosynthesis, nitrogen‐use efficiency, water‐use efficiency, and other gas exchange processes in the alpine tundra. Model simulations suggest that dry and wet meadow plant species do not significantly respond to changes in the volumetric soil moisture content but are sensitive to variation in foliar nitrogen content. In addition, model simulations indicate that dry and wet meadow species have different maximum rates of assimilation (normalized for leaf nitrogen content) because of differences in leaf temperature. These differences arise from the interaction of plant height and the abiotic environment characteristic of each plant community. The leaf temperature of dry meadow species is higher than wet meadow species and close to the optimal temperature for photosynthesis under current conditions. As a result, 2°C higher air temperatures in the future will likely lead to declines in dry meadow species’ carbon assimilation. On the other hand, a longer and warmer growing season could increase nitrogen availability and assimilation rates in both plant communities. Nonetheless, a temperature increase of 4°C may lower rates of assimilation in both dry and wet meadow plant communities because of higher, and suboptimal, leaf temperatures.

## 1 INTRODUCTION

The heterogeneous topography of mountainous ecosystems and subsequent differences in wind and radiation give rise to dry and wet meadow plant communities (Billings & Bliss, 1959; Choler, Michalet, & Callaway, 2001; Isard, 1986; Kikvidze et al., 2005; Litaor, Williams, & Seastedt, 2008; Sardinero, 2000; Scherrer & Korner, 2011; Walker, Theodose & Webber 2001). Dry meadow plant communities inhabit dry and warm environments, while wet meadow plant communities inhabit wet and cool environments (Isard, 1986; Litaor et al., 2008; Scherrer & Korner, 2011). In addition to occupying different abiotic niches, dry and wet meadow plant communities have species with distinct leaf trait assemblages (Choler, 2005; Spasojevic & Suding, 2012) that influence the rate of resource use and the relative performance of species under various physical conditions (Aerts & Chapin III, 2000; de Bello et al., 2013; Chapin III, Autumn, & Pugntairet, 1993; Soudzilovskaia et al., 2013; Suding et al., 2008). Dry meadow plant species have low leaf nitrogen content (0.8–2.0 g senescent plant N/m^2^) and low growth rates (84–198 g senescent plant biomass/m^2^) (Fisk, Schmidt, & Seastedt, 1998). According to the leaf economics spectrum (LES) (Diaz, Bradley, & Ning, 2014; Diaz et al., 2016; Reich, 2014; Wright et al., 2004; Zhao, Ali, & Yan, 2016), these leaf trait values enable resource conservation and so the dry meadow plant community has a “conservative” strategy. On the other hand, wet meadow plant species have high leaf nitrogen content (2.2–3.0 g senescent plant  N/m^2^) and high growth rates (230–309 g senescent plant biomass/m^2^) (Fisk et al., 1998), which increase resource acquisition. Thus, the wet meadow leaf trait assemblages is “acquisitive” under the LES. In this paper, we address the question of how abiotic factors and leaf trait assemblage characteristic of conservative and acquisitive strategies limit productivity in dry and wet meadow plant communities.

Productivity in tundra ecosystems is broadly limited by a combination of physical and nutrient controls (Bliss, 1962; Bowman & Fisk, 2001; Chapin III, 1987; Fan, Neff, & Wieder, 2016; Farrer et al., 2015). Seasonal changes in temperature limit productivity to a short growing season in the alpine tundra (Billings, 1974; Bliss, 1962; Walker et al., 1999; Wipf, Stoeckli, & Bebi, 2009). At the same time, plant communities are differentially limited by the volumetric soil moisture content (hereafter referred to as soil moisture content) due to the heterogeneous distribution of snowpack across the tundra (Billings & Bliss, 1959; Farrer et al., 2015; Greenland, 1989; Isard, 1986; Litaor et al., 2008; Natali, Schuur, & Rubin, 2012; Scherrer & Korner, 2011; Taylor & Seastedt, 1994). In addition, alpine plant communities are either primarily nitrogen‐limited or co‐limited by nitrogen and phosphorus as a result of cold temperatures and rocky soils (Bowman, Murgel, Blett, & Porter, 2012; Bowman, Theodose, Schardt, & Conant, 1993; Seastedt & Vaccaro, 2001; Soudzilovskaia, Onipchenko, Cornelissen, & Aerts, 2005). Although the patterns of limitation to plant growth are broadly understood, there is less information on the specific mechanisms that generate limitations to productivity (in time and space) and how these mechanisms differ across plant communities. A mechanistic understanding of plant productivity will improve predictions of plant community response to environmental changes in the alpine tundra ecosystem. Point‐ and ecosystem‐scale biogeochemical models can be used to explore the environmental drivers and seasonal trends in energy, water, and nutrient limitations in alpine flora (Fan et al., 2016; Wieder, Knowles, Blanken, Swenson, & Suding, 2017). However, these models do not include the photosynthetic mechanisms that influence the rate at which CO_2_ diffuses into the chloroplast and H_2_O diffuses out of the stomata. Instead, quantitative models of photosynthesis can be used to understand the leaf‐level drivers of carbon fixation and transpiration.

Photosynthesis models show how individual leaf traits influence rates of carbon assimilation, such as the leaf nitrogen and phosphorus content, specific leaf area (Walker et al., 2014; Wohlfahrt et al., 1999), and stomatal structure (de Boer et al., 2011). In addition, photosynthesis models demonstrate how abiotic factors, such as soil moisture content (Manzoni, Vico, Palmroth, Porporato, & Katul, 2013; Tanaka, Kosugi, & Nakamura, 2002), atmospheric carbon dioxide (Vico, Manzoni, Palmroth, Weih, & Katul, 2013), and leaf temperature (Lenz et al., 2010), regulate maximum rates of photosynthesis. Here, we use a photosynthesis model to test how leaf nitrogen content, leaf height above ground, leaf size, and leaf chlorophyll content interact with soil moisture content and air temperature to limit productivity in dry and wet meadow plant communities. Specifically, we simulate rates of assimilation for plant communities at the Niwot Ridge Long Term Ecological Research (LTER) site. Climate records indicate a trend toward longer growing seasons in mountainous regions like Niwot Ridge (Stewart, 2009; Stewart, Cayan, & Dettinger, 2004; Vaughan, 2013). In addition to a shorter winter, alpine environments in the Western United States are expected to have warmer springs and summers (Diaz et al., 2014; Diaz & Eischeid, 2007; McGuire, Nufio, Bowers, & Guralnick, 2012; Pepin et al., 2015), which may increase evaporation and lead to drier soil conditions at the peak of the growing season (Wipf, Gottfried, & Nagy, 2013). In order to understand present and future limitations to leaf‐level assimilation, we use an empirically parameterized and validated photosynthesis model to simulate plant community productivity in the current environment and compare it to productivity in an environment with lower soil moisture content, higher temperatures, and a longer growing season—that is, an extended summer.

## 2 METHODS

We simulated rates of assimilation for an average dry and wet meadow plant community at the Niwot Ridge LTER site by combining and expanding upon the Gaastra (1959), Farquhar, Caemmerer, and Berry (1980), and Ball, Woodrow, and Berry (1987) models of carbon assimilation and stomatal conductance. In addition, we outputted the instantaneous water‐ and nitrogen‐use efficiency (WUE and NUE) which, respectively, indicate how efficiently plants use limited water and nitrogen for productivity (Field, Merino, & Mooney, 1983; Field & Mooney, 1986; Schlesinger & Bernhardt, 2013b). Notably, we derived an empirically based model of leaf temperature as a function of leaf height. We also derived semi‐empirical equations that relate leaf nitrogen content and soil moisture content to maximum rates of assimilation. The model included abiotic and leaf trait parameters specific to the wet and dry meadow plant communities at Niwot Ridge (Table 1). Model simulations were tested against the best available empirical data obtained at Niwot Ridge during the peak of the growing season. After validating the model, we performed a series of model experiments to evaluate how leaf traits and environmental conditions affect dry and wet meadow species’ productivity**.**

**Table 1:** Average abiotic and leaf trait parameters that are specific to the dry and wet meadow species during the height of a typical growing season at Niwot Ridge (15 July–15 August). Mean values given. Standard deviations in parentheses. All parameters are adjusted for the influence of elevation

Acronym	Definition	Units	Dry meadow species values	Wet meadow species values	References
vwc	Midsummer volumetric soil moisture content	m^3^ m^−3^	0.12	0.29	http://niwot.colorado.edu (2013 and 2014 average)
*t*	Midsummer maximum surface temperature	°C	17.5	12.5	Scherrer and Korner, (2011); http://niwot.colorado.edu (2013 and 2014 average)
*z*	Soil depth	m	0.2	0.4	http://niwot.colorado.edu
chl	Leaf chlorophyll content	μmol Chl m ^−2^	396 (24)	476 (29)	Spasojevic et al. (2013)
ht	Leaf height	cm	9.2 (1.5)	20.0 (3.1)	Spasojevic et al. (2013)
dia	Leaf diameter	cm	1.6 (0.9)	3.0 (1.2)	Spasojevic et al. (2013)
na	Leaf nitrogen content	g N m^−2^	2.5 (1.1)	6.3 (1.1)	Fisk (1995)

### 2.1 Model inputs

**Figure 1:**
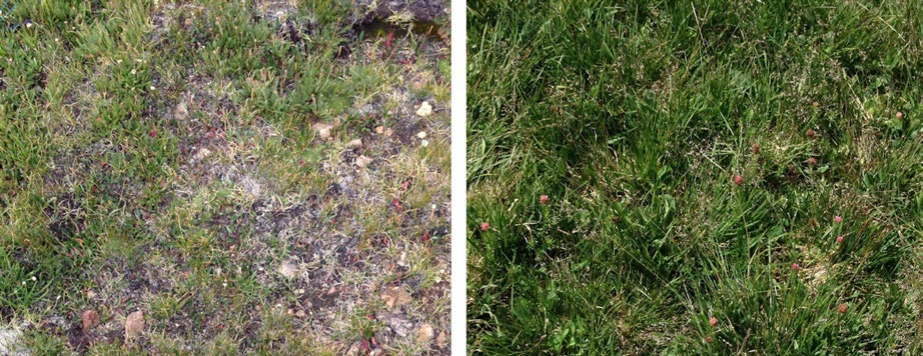
Photographs of the dry meadow (center) and wet meadow (right) plant communities at Niwot Ridge, CO. Photographs taken on 25 July 2018 by Kelsey Elwood

The Niwot Ridge LTER site is situated in the Colorado Front Range 35 km west of Boulder, Colorado, at an elevation of 3,500 m. The mean annual precipitation is 1,000 mm, 85% of which is snow, and the mean annual temperature is −3.8°C. Like other alpine sites, Niwot Ridge has a short, 2‐ to 3‐month‐long growing season with a mean temperature of 10°C (Knowles, 2015; http://niwot.colorado.edu). Additionally, Niwot Ridge contains plant communities that follow a moisture–temperature gradient largely determined by snow accumulation (Walker et al., ; Table 2). Figure 1 shows the two dominant plant communities that represent the end points of this gradient: the dry and wet meadow. The dry and hot environment of the dry meadow contains species with a conservative leaf trait assemblage, which we define as a low foliar nitrogen content, chlorophyll content, leaf area, and leaf height above the ground. On the other hand, the cool and wet environment of the wet meadow contains species with an acquisitive leaf trait assemblage, which we define as high foliar nitrogen content, chlorophyll content, leaf area, and leaf height above the ground (Fisk & Schmidt, 1995; Spasojevic, Bowman, Humphries, Seastedt, & Suding, 2013).

**Table 2:** Environmental parameters that are specific to the dry and wet meadow plant communities at Niwot Ridge

	Dry meadow environment	Wet meadow environment	References
Average summer temperature	10.86°C	6.43°C	Knowles, Blanken, and Williams (2015)
Average summer volumetric soil moisture content	0.16 m^3^ m^−3^	0.54 m^3^ m^−3^	Knowles et al. (2015)
Plant species	*Acomastylis rossii*, *Carex rupestris*, *Kobresia myosuroides*, *Selaginella densa*,* Trifolium dasyphyllum*	*Acomastylis rossii*, *Caltha leptosepala*, *Carex scopulorum*,* Deschampsia caespitosa*, *Salix arctica*	Bowman et al. (1995); Bowman (1994); Theodose and Bowman (1997), Bowman et al. (1993); http://niwot.colorado.edu

In order to validate modeled rates of maximum assimilation for the height of summer, we parameterized the model with physiological and environmental data that are characteristic of the alpine tundra biome (Table 3) and leaf traits and abiotic conditions that are specific to the plant communities (Table 1). We obtained parameter data from the literature and Niwot Ridge LTER database. We averaged daily soil moisture content and maximum air temperature data from 15 July to 15 August for the years 2013 and 2014 (http://niwot.colorado.edu). We adjusted dry and wet meadow surface temperatures so that they equal the average maximum air temperature (15°C) plus and minus 2.5°C, respectively (Scherrer & Korner, 2011; unpublished soil temperature data collected at Niwot Ridge). In the model, we used the total leaf nitrogen content for all plant species in the plant communities measured by Fisk (1995) on 1 August in 1992 and 1993. We also used the community‐weighted means of leaf chlorophyll content, leaf height, and leaf area which were measured from mid‐July to 1 August 2009 from Spasojevic et al. (2013). We derived leaf diameter from the leaf area, which we treated as a circle. Uncertainty is accounted for in some of the alpine tundra‐wide parameters (Table 3) by pulling 30 parameter values from a uniform distribution that has a range of ±20% of the parameter value. Model simulations additionally incorporated uncertainty in conservative and acquisitive leaf trait parameters (Table 1) by drawing 30 values from a normal distribution characterized by a mean and standard deviation.

**Table 3:** Physiological and environmental parameters that are similar across plant species at Niwot Ridge

Acronym	Definition	Units	Values	References
ra	Specific rubisco activity	μmol CO_2_ g Rub^−1^ s^−1^	20.7^a^	de Boer et al. (2011, Stinziano, Hüner, and Way (2015)
flnr	Fraction of total leaf nitrogen in rubisco	g N Rub g N leaf^−1^	0.1^a^	Field and Mooney (1986; Harrison et al. (2009; Poorter and Evans (1998; Vogan and Sage (2011
nr	Nitrogen content in rubisco molecule	g Rub g N Rub^−1^	6.25^b^	de Boer et al. (2011; Niinemets and Tenhunen, (1997; Poorter and Evans, (1998; Stinziano et al, (2015)
qeff	Efficiency of utilization of absorbed quanta	electrons	0.32^a^	Bjorkman (1981)
PAR	Midsummer average photosynthetically active radiation	μmol m^−2^ s^−1^	2000^a^	Bowman et al. (1995)
rh	Relative humidity	KPa kPa^−1^	0.5^b^	http://niwot.colorado.edu
fc	Field capacity (Minimum volumetric soil moisture content)	m^3^ m^−3^	0.08^a^	Saxton and Rawls (2006)
*M*_w_	Molarity of water	mol/L	55.6^b^	
*u*	Windspeed	m/s	5.0^b^	http://niwot.colorado.edu
*C*_a_	Ambient CO_2_	μmol CO_2_ mol air^−1^	405^b^	https://www.esrl.noaa.gov
O_2_	Ambient O_2_	μmol O_2_ mol air^−1^	210,000^b^	Schlesinger and Bernhardt (2013a)
*t*_oc25_	ratio of turnover number for oxygenase to carboxylase	unitless	0.21^b^	Farquhar et al. (1980)
*K*_c25_	Michaelis–Menten Kinetic coefficient for CO_2_ (25°C)	Pa	30^a^	Bonan (2008b)
*K*_o25_	Michaelis–Menten Kinetic coefficient for O_2_ (25°C)	Pa	30,000^a^	Bonan (2008b)
*e*_Kc_	Relative activation energy for K of CO_2_	J mol^−1^	80,500.0^a^	Medlyn, Dreyer, et al. (2002)
*e*_Ko_	Relative activation energy for K of O_2_	J mol^−1^	14,500.0^a^	Medlyn, Dreyer, et al. (2002)
*e*_tau_	Relative activation energy for K of Tau	J mol^−1^	−29,000.0^a^	Medlyn, Dreyer, et al. (2002)
*h*_d_	Enthalpy term	J mol^−1^	200,000.0^b^	Medlyn, Loustau, et al. (2002)
*e*_v_	Activation energy of carboxylation	J mol^−1^	55,000.0^a^	Medlyn, Loustau, et al. (2002)
*e*_j_	Activation energy of electron transport	J mol^−1^	55,000.0^a^	Medlyn, Loustau, et al. (2002)
*j*_m_	Slope of *J*_max_ versus *V*_cmax_	electrons CO_2_^−1^	2.68^a^	Leuning (1997)
*t*_opt_	Optimum temperature for maximum carboxylation and electron transport	K	303.0^b^	Wohlfahrt et al. (1999)
*g*_0_	Ball–Berry stomatal conductance intercept parameter	mol H_2_O m^−2^ s^−1^	0.002^a^	Bonan (2008b)
*m*	Ball–Berry stomatal conductance slope parameter	unitless	9^a^	Bonan (2008b)
*a*	Conversion coefficient between stomatal conductance to H_2_O and CO_2_	unitless	1.6^b^	Lambers et al. (2008)
*b*	Conversion coefficient between boundary layer conductance to H_2_O and CO_2_	unitless	1.37^b^	Lambers et al. (2008)
*D*_b_	Conversion coefficient between boundary layer conductance in m/s to mol m^−2^ s^−1^	unitless	27^b^	Bonan (2008a); Dingman (2014)

a Model is run with ±20% uncertainty in these parameter values.

b These parameter values are assumed to be relatively certain. All parameters are adjusted for the influence of elevation.

**Figure 2:**
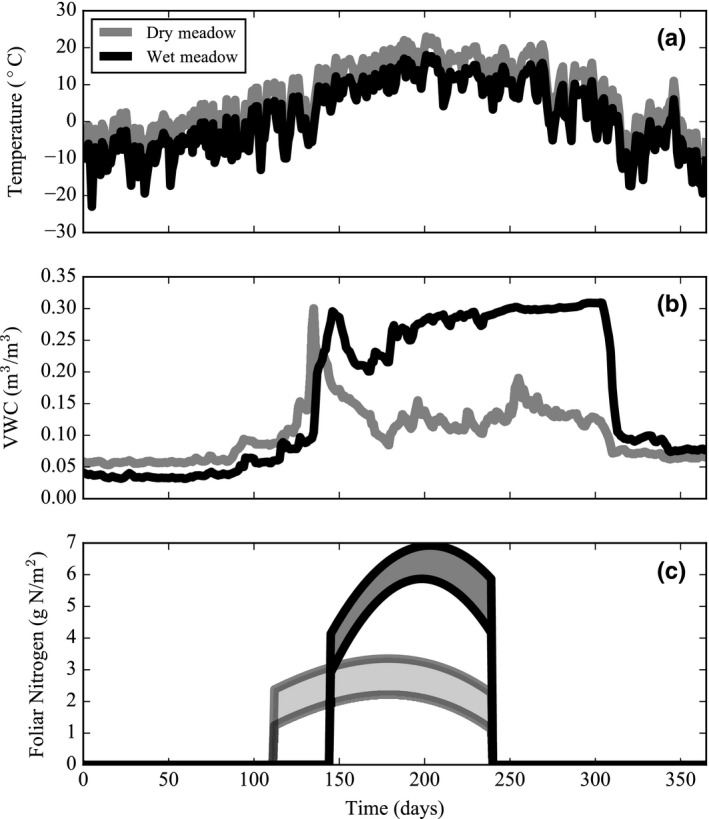
Environmental inputs for dry and wet meadow species during a typical growing season. (a) The 2014 time series of maximum temperatures in plant communities at Niwot Ridge (http://niwot.colorado.edu). In order to account for differences in surface temperature between plant communities, we adjusted the air temperature time series so that dry and wet meadow temperatures were 2.5°C higher and lower, respectively, than the recorded maximum temperatures (Scherrer & Korner, 2011; http://niwot.colorado.edu). (b) The 2013 time series of volumetric soil water (moisture) content in plant communities at Niwot Ridge (http://niwot.colorado.edu). (c) Models of daily leaf nitrogen content generated from observations of foliar nitrogen in Niwot Ridge plant communities taken at the beginning, middle, and end of the 1992 and 1993 growing seasons (Fisk, 1995)

In order to simulate inter‐ and intra‐annual changes in environmental parameters (for the simulation experiments), we used a time series of soil moisture content, air temperature, and foliar nitrogen content (Figure 2). We parameterized the model with daily values of maximum air temperatures in 2014 and soil moisture content in 2013 (Figure 2a,b). Unlike the other leaf traits, leaf nitrogen content reflects the distinct soil nitrogen content of the dry and wet meadow (Aerts & Chapin III, 2000; Bowman & Conant, 1994; Fisk & Schmidt, 1995). Similar to trends in leaf nitrogen content, Bowman, Bahn, and Damm (2003) observed higher rates of mineralized nitrogen in the wet meadow as compared to the dry meadow. To accommodate plasticity in leaf nitrogen, we developed a time series of leaf nitrogen content using leaf nitrogen data (mean and standard deviation) obtained during the start, middle, and end of the growing season at Niwot Ridge (Fisk, 1995; Figure 2c). In order to capture the initial increase of leaf nitrogen at the onset of the growing season and the decline in leaf nitrogen during senescence (Fisk et al., 1998; Jaeger III, Monson, Fisk, & Schmidt, 1999), we forced the leaf nitrogen content to zero prior to the start and following the end date of a typical growing season (Supporting Information Table S1).

### 2.2 Model equations

To calculate assimilation (*A*), we solved a system of equations following Baldocchi (1994)’s approach, which combines Farquhar et al. (1980)’s model of assimilation (Equation 1) with Ball et al. (1987)’s (Equation 2) and Gaastra (1959)'s (Equation 3) models of stomatal conductance.

(1) $$A=\text{min}\left(\frac{V_{cmax}C_{c}}{C_{c}+K_{c}\left(1+\frac{\left[O_{2}\right]}{K_{o}}\right)},\frac{JC_{c}}{4C_{c}+8\Gamma^{*}}\right)\left(1-\frac{\Gamma^{*}}{C_{c}}\right)$$

(2) $$g_{s}=\frac{m\cdot A\cdot rh}{C_{s}}+g_{0}$$

(3) $$A={(C}_{a}-C_{c})/\left(\left(1/g_{b}\right)+\left(1/g_{s}\right)+\left(1/g_{m}\right)\right)$$

We additionally included a term for mesophyll conductance, which is no longer considered infinite (Lambers, Chapin, & Pons, 2008; Singsaas, Ort, & Delucia, 2003). Multiple factors govern mesophyll conductance, such as the concentration of carbonic anhydrase, cell wall thickness, aquaporins, and chloroplast distribution and surface area (Field & Mooney, 1986; Flexas, 2012; Lambers et al., 2008). However, it is uncertain how these factors independently and collectively govern mesophyll conductance, so we set mesophyll conductance equal to stomatal conductance in the model (Lambers et al., 2008). Equation (4) shows the final equation used to calculate the rate of assimilation for dry and wet meadow plant communities as well as the variable definitions (Equations (4.1), (4.2), (4.3), (4.4), (4.5), (4.6), (4.7), (4.8), (4.9); Tables 1, 3; Supporting Information Equations S1–S13).

(4) $$0=A^{3}\cdot X1+A^{2}\left(X2+a_{2}\cdot X4-a_{1}\cdot X1\right)+A\left(X3+a_{2}.X5-a_{1}\cdot X2+a_{1}\cdot X4\cdot \Gamma^{*}\right)+\left(-a_{1}\cdot X3+X5{\cdot a}_{1}\cdot \Gamma^{*}\right)$$

(4.1) $$X1=-a\cdot m\cdot rh\cdot g_{b}+a\cdot g_{0}+2g_{b}$$

(4.2) $$X2=C_{a}\cdot a\cdot m\cdot rh\cdot g_{b}^{2}-a\cdot g_{0}{\cdot g}_{b}\cdot C_{a}-C_{a}\cdot a\cdot g_{0}\cdot g_{b}-2\cdot C_{a}\cdot g_{b}^{2}$$

(4.3) $$X3=C_{a}^{2}\cdot a\cdot g_{0}\cdot g_{b}^{2}$$

(4.4) $$X4=a\cdot m\cdot rh\cdot g_{b}^{2}-a\cdot g_{0}\cdot g_{b}$$

(4.5) $$X5=C_{a}\cdot a\cdot g_{0}\cdot g_{b}^{2}$$

(4.6) $$a_{1c}=V_{cmax}$$

(4.7) $$a_{2c}=K_{c}\left(1+\frac{O_{2}}{K_{o}}\right)$$

(4.8) $$a_{1j}=\frac{J}{4}$$

(4.9) $$a_{2j}=2\Gamma^{*}$$

The parameters: *V*_cmax_, *J*, *Γ* ^*^, *K*_c_, and *K*_o_ as well as the leaf vapor pressure deficit (vpd*; *see below) are a function of leaf temperature (Supporting Information Equations S6 and S7). In order to solve for leaf temperature, we plotted the difference between air and leaf temperature as a function of leaf height above the ground using data from Korner and Cochrane (1983). We then performed a linear least‐squares regression on the available data and derived leaf temperature (*t_l_*) as a function of leaf height (ht) and air temperature (*t*) (Equation 5; Supporting Information Figure S1). In this equation, an incremental increase in air temperature increases the leaf temperature by that same increment, while an increase in leaf height proportionally decreases the leaf temperature.

(5) $$t_{l}=t+\left(18-0.4\cdot ht\right)$$

We capped the rate of assimilation at a maximum threshold determined from an empirical relationship between foliar nitrogen and biomass production. We performed a linear least‐squares regression to derive an equation between leaf nitrogen and carbon content where the value of foliar carbon depends on the leaf nitrogen content and ranges from zero to one (Fisk, 1995). We then multiplied the *absolute* maximum rate of assimilation (26 μmol CO_2_/m^2^s in ideal conditions, that is, fertilized and irrigated under full sunlight and 20°C; Bowman, Theodose, & Fisk, 1995) by this linear model of foliar carbon in order to generate a maximum rate of assimilation, *A*_max_, for a species with a given leaf nitrogen content (na) (Equation 6).

(6) $$A_{max}=26\cdot \left(0.11na+0.03\right)$$

We additionally capped the rate of assimilation by the maximum rate of transpiration, which we determined from the soil moisture content. We calculated transpiration (*T*) using the vapor pressure deficit (vpd) and stomatal conductance (*g*_s_) of the leaf (Palmroth et al., 2013; Lambers et al., 2008; Manzoni et al., 2013; Supporting Information Equations S14–S17; Equation 7).

(7) $$T=\frac{g_{s}\cdot vpd}{a}$$

When transpiration exceeded the available soil moisture content (total soil moisture content in the soil minus the field capacity), we set transpiration equal to the available soil moisture content measured at that time step and solved for assimilation. In order to cap transpiration at this maximum value of soil moisture content, we converted both variables into units of L/m^2^ (Supporting Information Equations S18 and S19).

Finally, we used model values of assimilation and transpiration to calculate instantaneous NUE (Equation 8) and WUE (Equation 9) (Field & Mooney, 1986; Lambers et al., 2008; Schlesinger & Bernhardt, 2013b).

(8) $$NUE=\frac{A}{na}$$

(9) $$WUE=\frac{A}{T}$$

### 2.3 Model outputs

#### 2.3.1 Validation

The model outputted the rate of assimilation, NUE, and WUE for dry and wet meadow species during the height of a growing season (mid‐July through mid‐August). Model simulations were tested against the best available empirical data obtained at Niwot Ridge during the peak of the growing season. To validate instantaneous rates of assimilation, we calculated the average growth rates of dry and wet meadows from 2011–2014 as the ratio between peak carbon biomass and the number of days since the first snow‐free date when temperatures were above zero for three consecutive days (http://niwot.colorado.edu). We validated the simulated NUE and WUE with empirically derived NUE and WUE data (Bowman et al., 1995; Fisk et al., 1998). We validated the simulated NUE for both the dry and wet meadows. However, in order to test WUE simulations, we simulated moist meadow WUE rather than wet meadow WUE because the only WUE data available for Niwot Ridge are for dry and moist meadow plant communities. The moist meadow plant community was an appropriate test case because it contains a unique suite of plant species with leaf trait values that are similar to the wet meadow plant community and abiotic conditions that differ from the dry meadow plant community (Supporting Information Table S2). In the experiments (see following section), we reverted back to simulating dry and wet meadow WUE because the dry and wet meadows represent the two extremes of the alpine tundra: conservative and acquisitive leaf trait assemblages and drought and saturated abiotic conditions. The model also outputted stomatal conductance of CO_2_ and H_2_O as well as rates of transpiration; we validated these model variables with data from Niwot Ridge.

#### 2.3.2 Experiments

In the first model experiment, we explored how individual leaf traits and abiotic conditions affect rates of assimilation. For both plant communities, we modeled the rate of assimilation, NUE, and WUE over the course of a growing season. We then varied each mean leaf trait characteristic of dry and wet meadow plant communities by plus or minus one standard deviation and recorded the change in the simulated rate of assimilation. During this analysis, all other parameters remained at a constant mean value. Third, we simulated the rate of assimilation as a function of soil moisture content, temperature, and leaf nitrogen content while all other parameters remained at a constant value characteristic of the moist meadow. Finally, we explored how leaf traits and environmental variables interactively affect rates of assimilation. We simulated assimilation for an acquisitive and conservative leaf trait assemblage over the course of a growing season in both a dry and wet meadow environment. To capture site differences in foliar nitrogen content as shown by Bowman (1994), Fisk and Schmidt (1995), Bowman et al. (1995), and Fisk et al. (1998), we increased the foliar nitrogen content of alpine species with both conservative and acquisitive leaf trait assemblages when they occupied the wet meadow and decreased the foliar nitrogen content when they occupied the dry meadow.

In the second model experiment, we explored how three climate change scenarios impact assimilation in dry and wet meadow plant communities. In the first scenario, we simulated assimilation over the course of a growing season that has lower peak‐season soil moisture content, hotter air temperatures, and a longer period allotted for growth (i.e., an extended summer). In the second scenario, we simulated a longer growing season without changing the temperature or the soil moisture content, and in the third scenario, we simulated hotter temperatures without changing the growing season length or the soil moisture content. We did not include a scenario where we only decreased the soil moisture content because we found that soil moisture content does not limit productivity (see Section 3). For these scenarios, we increased temperatures by 2–2.5°C based on temperature data from a hot growing season in 2012 at Niwot Ridge (http://niwot.colorado.edu). We extended the onset of the growing season (i.e., the early‐season rapid increase in leaf nitrogen and soil moisture content) by 30 days because snow depth data indicate that snowmelt occurred a month earlier in 2012 as compared to the average snowmelt date (Supporting Information Table S1). Finally, we decreased the soil moisture content by 10% from mid‐June to mid‐September because the average soil moisture content across plant communities was 10% lower in 2012 as compared to a typical growing season in 2013 (http://niwot.colorado.edu). In both experiments, the time series outputs were smoothed using a Savitzky–Golay convolution method which fits successive subsets of adjacent points to a polynomial using linear least squares. This smoothing method reduced noise in the model output without distorting the overall seasonal trends in the model variables.

## 3 RESULTS

### 3.1 Model validation

Model prediction of instantaneous rates of assimilation in dry and wet meadow plant communities fell within the range of empirical measurements for an alpine biome (Table 4). Model results indicated that dry meadow species have a lower rate of assimilation (5 µmol CO_2_/m^2^ s) than wet meadow species (18 µmol CO_2_/m^2^ s). Similarly, wet meadow species had a higher measured daily growth rate (1.91 g C m^−2^ day^−1^) than dry meadow species (0.91 g C m^−2^ day^−1^) (Supporting Information Figure S2). Lastly, modeled assimilation increased as leaf nitrogen content increased from dry (2.45 g N/m^2^) to wet meadows (6.25 g N/m^2^). This is consistent with empirical measurements (Evans, 1989; Field & Mooney, 1986; Reich, Ellsworth, & Walters, 1998).

**Table 4:** The range of simulated values and empirical observations of key model variables. All empirically derived measurements are from plant species across the alpine tundra

	Units	Model values	Empirical values	References
Assimilation (A)	µmol CO_2_/m^2^s	2–26	1–22	Bowman et al. (1993), Bowman et al. (1995), Billings, Clebsch, and Mooney (1996)
Nitrogen‐use efficiency (NUE)	µmol CO_2_/g N s	1–3	10	Bowman et al. (1995)
Water‐use efficiency (WUE)	µmol CO_2_/mmol H_2_O	1–4	1–2	Bowman et al. (1995)
Stomatal conductance to CO_2_ (*g*_s_)	mmol CO_2_/m^2^s	40–500	400–1,100	Bowman et al. (1995)
Stomatal conductance to water (*g*_s_/1.6)	mmol H_2_O/m^2^s	30–300	50–370	Spasojevic and Suding (2012)
Transpiration (T)	mmol H_2_O/m^2^s	1–10	0–17	Bowman et al. (1995), Berkelhammer et al. (2016)**; ** http://niwot.colorado.edu

The simulated instantaneous NUE across dry and wet meadow plant species was lower than the 10 µmol CO_2_/g N s recorded for *Kobresia myosuroides*, an alpine tundra plant common to dry meadows (Table 4). Overall, modeled NUE was lower in dry meadow species (1.9 µmol CO_2_/g N s) and higher in wet meadow species (2.9 µmol CO_2_/g N s). Similar to the simulated trend in instantaneous NUE across plant communities, Fisk et al. (1998) observed that the integrated NUE (g biomass/g nitrogen of senescent plant material at the close of the growing season; Berendse & Aerts, 1987; Chapin III, Matson, & Vitousek, 2012) was significantly higher in the wet meadow (88 g biomass g N^−1^) than the dry meadow (72 g biomass g N^−1^) (Supporting Information Figure S3).

Simulated values of instantaneous WUE were within the range observed for dry and wet meadow species at Niwot Ridge (Table 4). Modeled WUE was higher in wet meadow species (3.1 µmol CO_2_/mmol H_2_O) and lower in dry meadow species (1.5 µmol CO_2_/mmol H_2_O). Like wet meadow species, moist meadow species’ simulated WUE (2.5 µmol CO_2_/mmol H_2_O) was higher than dry meadow species’ WUE. However, empirical measurements indicate that instantaneous WUE is constant (1.5 µmol CO_2_/mmol H_2_O) across Niwot Ridge dry and moist meadow plant communities (Bowman et al., 1995; Supporting Information Figure S4).

Simulated values of stomatal conductance of CO_2_ were in the lower range of values observed for dry and moist meadows at Niwot Ridge, while simulations of stomatal conductance of H_2_O were in agreement with the range of values observed at Niwot Ridge (Table 4). Modeled stomatal conductance of CO_2_ increased proportionally with rates of assimilation (Supporting Information Figure S5). A similar positive relationship between assimilation and stomatal conductance is apparent in the literature (von Caemmerer & Farquhar, 1981; Farquhar & Sharkey, 1982). Modeled values of transpiration were in between the observed value of transpiration averaged across Niwot Ridge (~1.0 mmol H_2_O/m^2 ^s; http://niwot.colorado.edu; Table 4), which includes rock, snow, and water surfaces, and the observed values of transpiration for the dry and moist meadow plant communities at Niwot Ridge (~13.5 mmol H_2_O/m^2 ^s; Bowman et al., 1995; Table 4).

### 3.2 Model experiments

**Figure 3:**
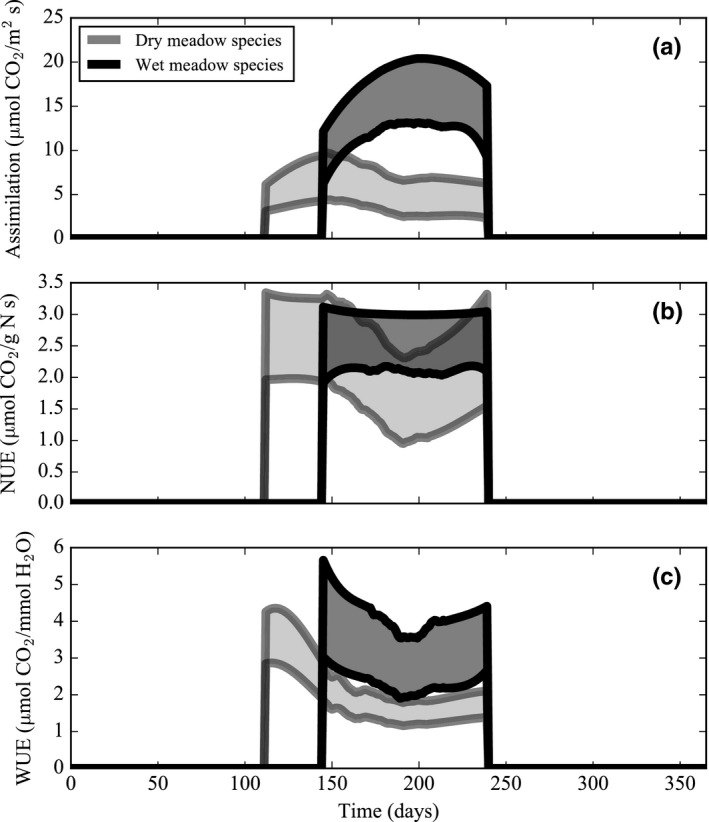
Simulated assimilation rates (a), nitrogen‐use efficiency (b), and water‐use efficiency (c) for dry and wet meadow species over the course of an average growing season. Simulation outputs are smoothed using a Savitzky–Golay filter over the time series. The shaded area shows the range of 30 simulated values when the model is run with parameter uncertainty during each daily time step

**Figure 4:**
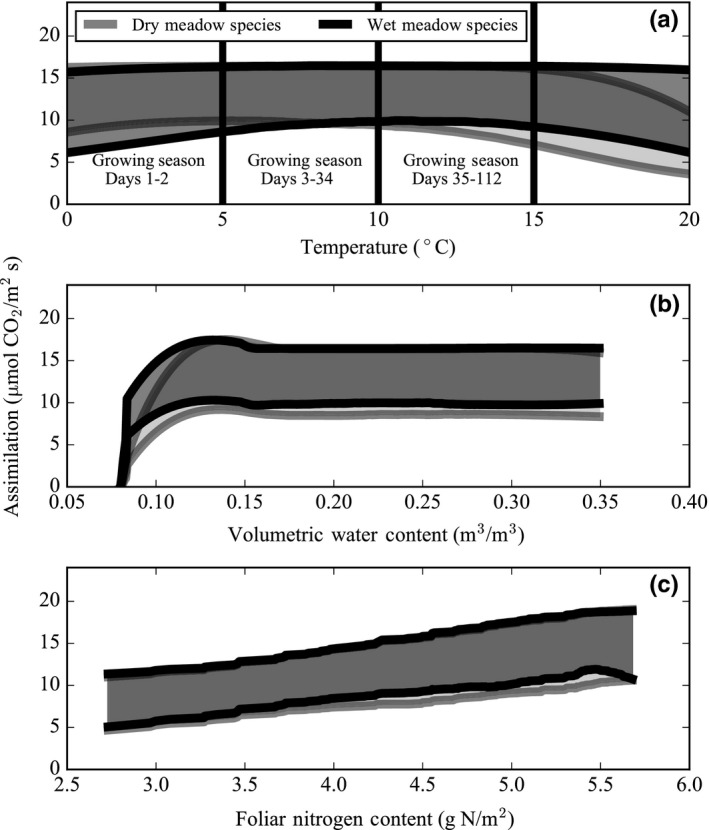
Simulated assimilation rates of dry and wet meadow species as a function of temperature (a), volumetric soil water (moisture) content (b), and foliar nitrogen content (c). When isolating an environmental variable, all other environmental variables remain at a constant growing season average value characteristic of the moist meadow. Simulation outputs are smoothed using a Savitzky–Golay filter over the time series. The shaded area shows the range of 30 simulated values when the model is run with parameter uncertainty during each daily time step

**Figure 5:**
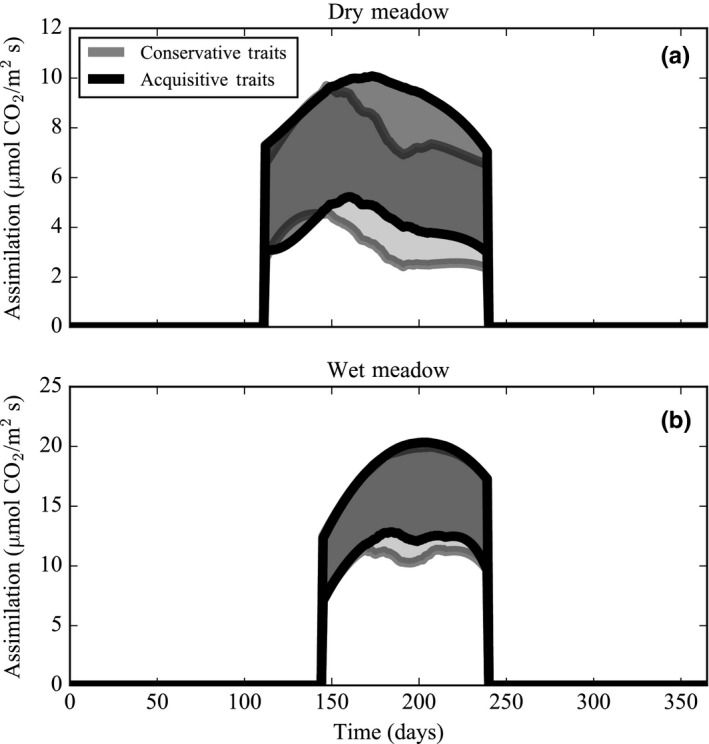
Simulated assimilation rates of conservative and acquisitive leaf trait assemblages in a dry meadow environment (a) and a wet meadow environment (b) over the course of an average growing season. Simulation outputs are smoothed using a Savitzky–Golay filter over the time series. The shaded area shows the range of 30 simulated values when the model is run with parameter uncertainty during each daily time step

In the first model experiment, the dry meadow species’ rates of assimilation were consistently lower than the wet meadow species (Figure 3a). Of the conservative and acquisitive leaf traits, dry and wet meadow species’ assimilation rates were most responsive to changes in the leaf nitrogen content. A change in leaf height also affected dry meadow species’ rate of assimilation (Table 5). Simulated rates of assimilation for dry and wet meadow species followed seasonal trends in the leaf nitrogen content; both communities increased assimilation in response to increasing foliar nitrogen (Figures 3a and 4c). Dry and wet meadow species’ rates of assimilation also appeared to increase in response to a peak in soil moisture content at the onset of the growing season (Figure 3a); however, soil moisture content governed assimilation only when soil moisture content was lower than ~0.1 m^3^ m^−3^ (Figure 4b). Rather, trends in assimilation were tightly coupled with temperature. As the growing season progressed, dry meadow species’ assimilation rates gradually increased from April to May when maximum temperatures were low (~0–8°C) and then decreased below wet meadow species’ assimilation rates for the remainder of the growing season. On the other hand, wet meadow species’ assimilation rates steadily increased in June toward a peak in mid‐July when maximum temperatures were high (~12–20°C) (Figures 3a and 4a). Similarly, as temperatures escalated, the dry meadow species’ NUE dipped in the middle of the growing season, while the wet meadow species’ NUE was constant and high throughout the growing season (Figure 3b). WUE followed trends in temperature but not in soil moisture content and decreased in both plant communities over the course of the growing season (Figure 3c). Species with a conservative leaf trait assemblage had lower rates of assimilation in both the dry and wet meadow environments. In the dry meadow environment, alpine species (with both types of leaf trait assemblages) had lower rates of assimilation at the height of summer than in the wet meadow environment (Figure 5).

**Table 5:** Sensitivity analysis of leaf trait parameters given in Table 1

∆ Assimilation (%; μmol CO_2_/m^2^s)				
	Leaf nitrogen content	Leaf chlorophyll content	Leaf diameter	Leaf height
Dry meadow	44%; |2.2|	0%; |0|	0%; |0|	7%; |0.4|
Wet meadow	17%; |3.1|	0%; |0|	0%; |0|	0%; |0|

Note

The values shown in the table are the percent and absolute change in dry and wet meadow species’ assimilation when each trait is perturbed by ±the plant community‐weighted standard deviation.

**Figure 6:**
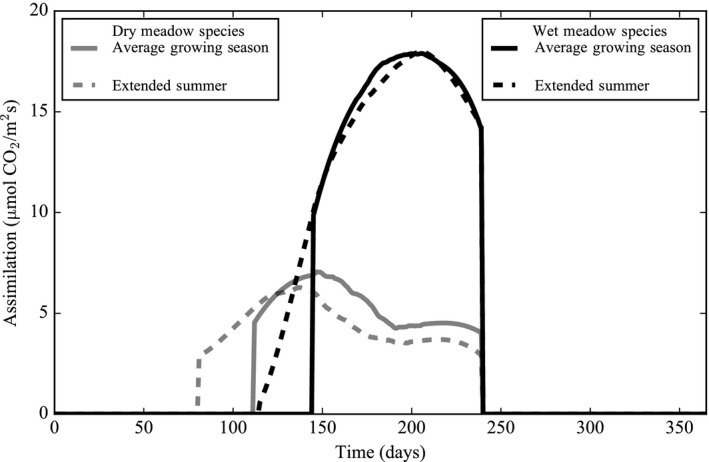
Simulated assimilation rates of dry and wet meadow species in an extended summer scenario, which includes warmer temperatures and a longer growing season, juxtaposed against an average growing season. Simulation outputs are smoothed using a Savitzky–Golay filter over the time series. The lines show the average of 30 simulated values when the model is run with parameter uncertainty during each daily time step

In the second model experiment, wet meadow species increased their total growing season assimilation, that is, cumulative assimilation, by 7% in an extended summer scenario. On the other hand, the dry meadow species’ cumulative assimilation increased by only 1% in the same scenario (Figure 6; Table 6). In the longer growing season scenario, cumulative assimilation increased in both dry and wet meadow species following the increase in leaf nitrogen content (Table 6). In this case, the percent change in assimilation was different between dry and wet meadow plant communities because the same absolute change in leaf nitrogen content equated to an unequal *percent* change in foliar nitrogen content. Under the hotter temperature scenario, cumulative assimilation decreased in both plant types. Dry meadow species decreased their cumulative assimilation by 17% while the wet meadow species decreased their assimilation by only 1%. In this scenario, the percent change in air temperature was the same between dry and wet meadow plant communities, so the differential response resulted from differences in either the leaf trait assemblage or environmental conditions between the plant communities (Table 6).

**Table 6:** Percent and absolute change in cumulative assimilation as a result of three different scenarios

Entire growing season (%;μmol CO_2_/m^2^)		Extended summer scenario	Longer growing season scenario	Hotter temperatures scenario
Dry meadow ∆ Assimilation	Conservative leaf traits	+1%; + 7	+18%; +119	−17%; −114
	Acquisitive leaf traits	+6%; +55	+12%; +96	−7%; −57
Wet meadow ∆ Assimilation	Conservative leaf traits	+0%; +10	+9%; +134	−8%; −121
	Acquisitive leaf traits	+7%; +107	+8%; +121	−1%; −27

Note

The extended summer scenario is a combination of the longer growing season and hotter temperatures scenarios.

## 4 DISCUSSION

The alpine tundra contains multiple plant communities with distinct abiotic environments and plant species. However, we focus our analysis only on the dry and wet meadow plant communities because they represent the two extremes of an alpine ecosystem: drought versus saturation and conservative versus acquisitive leaf trait assemblages. We assume that these plant communities show the greatest difference in rates of assimilation. Model simulations show the cumulative effect of abiotic and physiological controls on both plant communities’ assimilation rates, NUE, and WUE under current and projected climates. We have higher confidence in the validity of modeled assimilation rates and WUE than modeled trends in NUE. The lower confidence in the NUE simulation results is due to the paucity of data from Niwot Ridge and other alpine sites. Model simulations suggest that soil moisture content minimally affects plant assimilation in dry and wet meadow plant communities in large part because even in the driest portion of the growing season, there is sufficient water to support estimated rates of plant assimilation. On the other hand, current peak‐season air temperatures limit assimilation in the dry meadow. The same ambient air temperature, however, does not limit assimilation in the wet meadow because the wet meadow's *leaf* temperature differs from the dry meadow. As compared to wet meadow species, dry meadow species have hotter and less optimal leaf temperatures because of their short plant height and warm environment. This relationship held true when we modeled hotter temperatures in a future climate: dry meadow species decreased their rate of assimilation to a greater extent than wet meadow species. Temperature constraints on photosynthesis reduced dry meadow species’ NUE relative to wet meadow species. As a result, dry meadow species may be less equipped than wet meadow species to utilize an increase in total foliar nitrogen content during a longer and warmer growing season. We conclude that the different leaf temperatures of dry and wet meadow species could play an important role in determining the relative performance of these plant communities in the future.

### 4.1 Leaf‐level limitations to assimilation in the dry and wet meadow

Despite large differences in soil moisture content between the two plant communities examined here, model simulations indicate that seasonal changes in soil moisture content do not affect assimilation in alpine plant species (Figure 4). Assimilation and transpiration increase proportionally with soil moisture content only in the narrow zone between field capacity (0.08 m^3^ m^−3^) and a soil moisture content of 0.1 m^3^ m^−3^. For 90% of the dry meadow growing season and 100% of the wet meadow growing season, soil moisture content is above 0.1 m^3^ m^−3^ and this level is sufficient to support the estimated transpiration demands of plants in both communities. Moreover, both the dry meadow growing season average soil moisture content (0.16 m^3^ m^−3^) and the wet meadow growing season average soil moisture content (0.54 m^3^ m^−3^) are well above this threshold (Table 2). In order to reach a growing season average of 0.1 m^3^ m^−3^ threshold, the dry meadow would require a 50 mm decrease in average precipitation and the wet meadow would require a 380 mm decrease in precipitation. Although this result may seem counterintuitive given the names of the two communities (wet and dry meadow), it is consistent with findings from experimental manipulations at Niwot Ridge that show that plant communities respond to nitrogen addition but are unresponsive to water addition (Bowman, Gartner, Holland, & Wiedermann, 2006; Bowman et al., 1993, 1995; Gasarch & Seastedt, 2015). The lack of water limitation in the dry and wet meadow plant communities may be a result of their slow maximum rate of assimilation, which only requires a soil moisture content of 0.01 m^3^ m^−3^. Factors other than water downregulate rates of assimilation, such as the low leaf nitrogen content and cold temperatures characteristic of alpine tundra plant communities.

Although plant communities are mostly unaffected by seasonal changes in soil moisture content, model simulations indicate that seasonal changes in leaf temperature constrain the maximum rate of assimilation in dry and wet meadow communities (Figure 4). Observations indicate that the leaf temperature optimum of C3 plants ranges from 15–35°C and can vary between species growing under different environments (Chapin III et al., 1993; Lambers et al., 2008; Larcher, 1995). Temperatures above and below this threshold denature rubisco and limit photosynthesis (Medlyn, Dreyer, et al., 2002; Medlyn, Loustau, Loustau, & Delzon, 2002). Despite differences in abiotic conditions, dry and wet meadow plant communities both maximize photosynthesis when leaf temperatures range from 15 to 25°C. Bowman et al. (1995) also found that assimilation rates per unit of foliar nitrogen did not significantly differ between the dry and moist meadow environments at Niwot Ridge when leaf temperatures were held constant at 20°C.

Dry and wet meadow species do not equally respond to seasonal changes in the *ambient air temperature* of the alpine tundra because of differences in the *leaf temperature*, which ultimately determines the rate of assimilation. The average growing season leaf temperature differs between dry (33°C monthly average) and wet meadow (24°C monthly average) plant communities. Two factors, surface air temperature and plant height, interact to create different leaf temperatures in dry and wet meadow species. The first factor, surface air temperature, deviates from the ambient air temperature and is dissimilar between plant communities. Scherrer and Korner (2010) observed a surface air temperature difference as high as 8°C between mean ambient air temperature (2 m above the surface) and the mean surface temperature during July and August in the Swiss Alps and other alpine sites in Sweden and Norway. In the Swiss Alps and at Niwot Ridge, the dry meadow has warmer surface air temperatures throughout the growing season as compared to the wet meadow because the dry meadow receives more radiation and has less snowpack due to its southern aspect and windward position (Isard, 1986; Scherrer & Korner, 2011; https://niwot.colorado.edu). In both dry and wet meadow species, model results show that a 5°C higher surface air temperature (i.e., dry meadow physical environment relative to the wet meadow physical environment) increases the leaf temperature and reduces midsummer assimilation rates despite a simultaneous peak in leaf nitrogen content (Figure 5). The second factor, plant height, also modulates leaf temperature and varies between dry and wet meadow species. Leaves that are close to the ground (i.e., conservative leaf trait assemblage of dry meadow species) remain warmer during the growing season than leaves that are taller in stature (i.e., acquisitive leaf trait assemblage of wet meadow species) (Korner & Cochrane, 1983; Salisbury & Spomer, 1964). In our model, 10‐cm‐tall dry meadow species have leaves that are 5°C warmer than 20‐cm‐tall wet meadow species’ leaves. Model simulations show that species with a conservative leaf trait assemblage have lower rates of assimilation during the majority of the growing season as compared to species with an acquisitive leaf trait assemblage because of differences in the leaf height (Figure 5; Table 5). When the differences in surface air temperature and plant height are both taken into account, the optimal ambient air temperature ranges from ~8 to 18°C for tall wet meadow species and from ~ −3 to 8°C for short dry meadow species (Figure 4).

Similar to air temperature, leaf nitrogen limits assimilation in alpine plant species. However, unlike air temperature, dry and wet meadow species equally increase assimilation in response to increased leaf nitrogen content. Any variation in the modeled NUE is a function of leaf temperature rather than plant utilization of leaf nitrogen during photosynthesis (Figure 4). Therefore, observed differences in leaf nitrogen content, and the maximum rate of assimilation, can be attributed to plant physiology and environmental factors rather than leaf‐level NUE. At the beginning of the growing season, nitrogen storage accounts for 56%–100% of the foliar nitrogen requirement of fertilized and unfertilized plant communities at Niwot Ridge (Aerts & Chapin III, 2000; Bowman et al., 2003; Castle & Neff, 2013; Lipson, Bowman, & Monson, 1996; Mooney & Billings, 1960). During the remainder of summer, soil available nitrogen and plant uptake rates determine the foliar nitrogen content (Aerts & Chapin III, 2000; Chapin III, 1987; Fisk & Schmidt, 1995). The dry meadow is typically characterized by low rates of nitrogen mineralization and plant uptake of mineralized nitrogen, while the wet meadow has higher rates of mineralization and uptake (Bowman et al., 2003; Bowman & Conant, 1994; Fisk et al., 1998). Model results indicate that differences in the leaf nitrogen between plant communities as a result of these physiological and environmental mechanisms enable higher rates of assimilation in wet meadow species as compared to dry meadow species (Figure 5).

### 4.2 Alpine tundra species’ response to an extended summer

The differential response of wet meadow and dry meadow species to seasonal changes in air temperature may be amplified by climate change. Here, we focus our analysis on the month of July because empirical data suggest that the greatest increase in air temperature will occur during this summer month at Niwot Ridge (McGuire et al., 2012). If the observed trends continue, then in the next 30 years there will be a 1.2–4.5°C temperature increase in July maximum temperatures. Currently, the average maximum air temperature in July is 19 and 14°C in the dry and wet meadow environments, respectively. When the model factors in the effect of plant height and surface air temperature on leaf temperature, wet meadow species’ current leaf temperatures surpass the optimal 25°C for 14 days in July with a 28°C maximum temperature. In the dry meadow plant community, leaf temperatures surpass the optimal 25°C for the entire month of July and leaf temperatures reach as high as 37°C under present conditions. Model simulations indicate that a 2°C increase in July maximum temperatures may decrease dry meadow species’ assimilation to a greater extent than wet meadow species’ assimilation (Table 6). A 4°C increase in July temperature doubles the number of days that wet meadow species’ leaf temperatures surpass the optimal 25°C and leaf temperature reaches as high as 32°C. The same change in temperature more than doubles the number of days that dry meadow species’ leaf temperatures surpass 35°C and dry meadow leaf temperatures reach as high as 41°C. Although model results suggest that dry meadow species are generally more responsive to higher temperatures than wet meadow species, both plant communities will likely reduce their rates of assimilation in a 2–4°C warmer climate.

One major question about the impacts of rising temperature is how nitrogen availability responds to warmer temperatures. For example, a longer growing season accompanied by higher temperatures may increase available soil nitrogen content (Rustad et al., 2001). To a lesser extent, nitrogen deposition may also increase soil nitrogen in the alpine tundra and lead to higher foliar nitrogen in dry and wet meadow species (Bowman et al., 2006; Bowman & Steltzer, 1998; Sievering, Rusch, & Marquez, 1996). When we simulate higher leaf nitrogen content coupled with 2°C higher temperatures (i.e., the extended summer scenario), cumulative assimilation either increases by 7% in wet meadow species with an acquisitive leaf trait assemblage or increases by 1% in the dry meadow species with a conservative leaf trait assemblage (Table 6). Although a longer and warmer growing season may increase the foliar nitrogen content and assimilation rates of alpine species, if air temperatures increase by 4°C, as predicted by McGuire et al. (2012), both dry and wet meadow species may still reduce their overall rate of assimilation.

In addition to simulating an overall increase in leaf nitrogen content as a result of climate change, we also simulated rates of assimilation in the case where dry and wet meadow species have identical leaf nitrogen content. Bowman (1994) and Bowman et al. (1995) observed that dry and wet meadow plant communities have a high degree of foliar nitrogen plasticity in response to fertilization. For example, after fertilization, the dry meadow plant community had a similar foliar nitrogen content as the control wet meadow plant community. In addition, Bowman (1994) showed that dry meadow plant species increased their foliar nitrogen to a greater degree than wet meadow species in response to fertilization. Therefore, a future increase in mineralized nitrogen content in the dry meadow alone, or across the alpine tundra, may lead to dry and wet meadow communities having a similar leaf nitrogen content. When plant communities have identical foliar nitrogen content, the rate of assimilation is similar between dry and wet meadow species; in this scenario, the small difference in the rate of assimilation between plant communities is due to temperature (Figure 4). Increased nitrogen mineralization could, thus, contribute to interspecific competition between dry and wet meadow species (Theodose, Jaeger, Bowman, & Schardt, 1996) and shifts in species abundance in the alpine tundra (Elmendorf, 2012; Farrer et al., 2015; Soudzilovskaia et al., 2013; Spasojevic et al., 2013) because of differences in the leaf temperature.

### 4.3 Model limitations

The model does not include biotic or abiotic feedbacks to rates of assimilation in plant communities. For example, in dry and wet meadow plant communities, plant composition determines the community response to nitrogen additions (Gasarch & Seastedt, 2015). In the wet meadow, nitrogen fertilization increases the abundance of a dominant graminoid, Deschampsia cespitosa and decreases species diversity. Together, these factors reduce the wet meadow's production relative to the dry meadow. The dry meadow has a higher production response to nitrogen enrichment because the dominant sedge, Kobresia myosuroides, decreases as a result of fertilization (Bowman et al., 1993; Gasarch & Seastedt, 2015; Theodose & Bowman, 1997). Therefore, the rate of dry meadow assimilation in an extended summer may be higher than modeled assimilation rates, while the rate of assimilation in the wet meadow may be lower than the modeled assimilation rates. An example of an abiotic response that is not included in the model is how changes in temperature and soil moisture content affect the relative humidity—a key model variable. The dry meadow, with its low soil moisture content and higher temperatures, should have a lower relative humidity than the cooler wet meadow (Dingman, 2014). Modeled rates of assimilation would be improved if the relative humidity is adjusted for dry and wet meadow plant communities under current conditions and in a hotter and drier climate.

Model simulations do not account for acclimation of temperature optima within a species or how leaf traits other than leaf height affect the leaf temperature. Several studies indicate that the temperature optima of plant species shift upward under hotter temperatures to support a smaller abundance of more thermally stable enzymes (Badger, Bjorkman, & Armond, 1982; Berry & Bjorkman, 1980; Ferrar, Slatyer, & Vranjic, 1989). Furthermore, when subject to different environmental temperatures, plants can invest in alternative photosynthetic machinery that require different thermal optima, such as RuBP regeneration rather than rubisco carboxylation (Dreyer, Le Roux, Montpied, Daudet, & Masson, 2001; Hikosaka, 1997). In a light‐saturated environment, such as in the alpine tundra (Bowman & Fisk, 2001), alternative investments in light‐capturing photosynthetic machinery under suboptimal temperatures will still reduce assimilation. In addition, model simulations do not consider how other traits such as leaf size and shape (Givnish & Vermeij, 1976; Nicotra, Cosgrove, Cowling, Schlichting, & Jones, 2008; Smith, 1978) and stomatal evaporative cooling (Crawford, McLachlan, Hetherington, & Franklin, 2012; Radin, Lu, Percy, & Zeiger, 1994) contribute to leaf temperature. For example, species with warmer and/or larger leaves may transpire more water and have a lower leaf temperature than species with cooler and/or smaller leaves (Dingman, 2014; Givnish & Vermeij, 1976). Model predictions of assimilation in an extended summer would be improved by further research on temperature optima acclimation and leaf temperature regulation by multiple leaf traits in dry and wet meadow species.

Finally, the model is parameterized with data that spans 20 years which may cause errors in model validation and analysis. In particular, the leaf nitrogen content used in the model is taken from measurements made in 1995, while other leaf trait and environmental parameters come from the years 2013 and 2014. The NUE (assimilation/leaf nitrogen) and WUE (assimilation/transpiration) are not affected by different values of leaf nitrogen because assimilation and transpiration increase proportionally with the leaf nitrogen content. Given the strong coupling between leaf nitrogen content and environmental conditions (Aerts & Chapin III, 2000; Bowman & Conant, 1994; Fisk & Schmidt, 1995), the time discrepancy between these variables may reduce the accuracy of the modeled values of absolute rates of assimilation. Nonetheless, a time lag between parameter inputs should not affect the model analysis and the conclusions of this paper which pertain to how the changes in individual parameter inputs alter rates of assimilation between the two plant communities.

## 5 CONCLUSION

The results of this model study indicate that assimilation in dry and wet meadow species is strongly affected by foliar nitrogen content which in turn varies in response to differences in soil available nitrogen across the alpine tundra. In addition, these simulations indicate that assimilation rates in both the dry and wet meadow are not constrained by soil moisture content but are sensitive to leaf temperatures which regularly exceed optimum values in the dry meadow community. In a longer and hotter summer simulation, the taller average plant stature and cooler environment characteristic of the wet meadow lead to high rates of assimilation relative to the dry meadow. Despite possible increases in leaf nitrogen during an extended summer, 4°C higher air temperatures will likely decrease assimilation in both plant types as their leaf temperatures reach suboptimal levels. Although model results are subject to environmental parameters unique to the alpine tundra, this research shows the importance of leaf traits and the abiotic environment in governing the leaf temperature, which may ultimately determine the relative performance of plant species in a world characterized by rapid climate change.

## CONFLICT OF INTEREST

None declared.

## AUTHORS CONTRIBUTION

Katherine Wentz substantially contributed to the conception or design of the work and the acquisition, analysis, or interpretation of data for the work; drafted the work or revised it critically for important intellectual content; approved the final version to be published; agreed to be accountable for all aspects of the work in ensuring that questions related to the accuracy or integrity of any part of the work are appropriately investigated and resolved; and wrote the quantitative model and performed the analyses. Jason Neff substantially contributed to the conception or design of the work and the acquisition, analysis, or interpretation of data for the work; drafted the work or revised it critically for important intellectual content; approved the final version to be published; agreed to be accountable for all aspects of the work in ensuring that questions related to the accuracy or integrity of any part of the work are appropriately investigated and resolved; and helped in the development of the quantitative model and relevant analyses. Katharine Suding substantially contributed to the conception or design of the work and the acquisition, analysis, or interpretation of data for the work; drafted the work or revised it critically for important intellectual content; approved final version to be published; agreed to be accountable for all aspects of the work in ensuring that questions related to the accuracy or integrity of any part of the work are appropriately investigated and resolved; and provided data resources for parameterization and validation of the model. Both Katharine Suding and Jason Neff imparted integral background information about the subject material which informed the research question, analyses, and conclusions of the paper.

## Data Availability

No new data were produced in writing this paper. See in‐paper references for data sources used in the research.

## References

[ece34816-bib-0001] Aerts, R. , & Chapin, F. S. III (2000). The mineral nutrition of wild plants revisited: A re‐evaluation of processes and patterns. Advances in Ecological Research, 30, 1–67.

[ece34816-bib-0002] Badger, M. R. , Bjorkman, O. , & Armond, A. (1982). An analysis of photosynthetic response and adaptation to temperature in higher plants: Temperature acclimation in the desert evergreen *Nerium* *oleander* . Plant, Cell and Environment, 5(1), 85–99.

[ece34816-bib-0003] Baldocchi, D. (1994). An analytical solution for coupled leaf photosynthesis and stomatal conductance models. Tree Physiology, 14(7_9), 1069–1079. 10.1093/treephys/14.7-8-9.1069 14967671

[ece34816-bib-0004] Ball, J. T. , Woodrow, I. E. , & Berry, J. A. (1987). A model predicting stomatal conductance and its contribution to the control of photosynthesis under different environmental conditions. Progress in Photosynthesis Research, 4(5), 221–224.

[ece34816-bib-0005] Berendse, F. , & Aerts, R. (1987). Nitrogen‐use‐efficiency: A biologically meaningful definition? Functional Ecology, 1(1), 293–296.

[ece34816-bib-0006] Berkelhammer, M. , Noone, D. C. , Wong, T. E. , Burns, S. P. , Knowles, J. F. , Kaushik, A. , … Williams, M. W. (2016). Convergent approaches to determine an ecosystem’s transpiration fraction. Global Biogeochemical Cycles, 30(6), 933–951. 10.1002/2016GB005392

[ece34816-bib-0007] Berry, J. A. , & Bjorkman, O. (1980). Photosynthetic response and adaptation to temperature in higher plants in higher plants. Annual Review of Plant Physiology, 31, 491–543. 10.1146/annurev.pp.31.060180.002423

[ece34816-bib-0008] Billings, W. D. (1974). Adaptations and origins of alpine plants. Arctic and Alpine Research, 6(2), 129–142. 10.2307/1550081

[ece34816-bib-0009] Billings, W. D. , & Bliss, L. C. (1959). An alpine snowbank environment and its effects on vegetation, plant development and productivity. Ecology, 40(3), 388–397. 10.2307/1929755

[ece34816-bib-0010] Billings, W. D. , Clebsch, E. E. , & Mooney, H. A. (1996). Photosynthesis and respiration rates of Rocky Mountain alpine plants under field conditions. The American Midland Naturalist, 75, 34–44.

[ece34816-bib-0011] Bjorkman, O. (1981). Responses to different quantum flux densities In O. L Lange, P. S Nobel, Osmond C. B. & Ziegler H. (Eds.), Encyclopedia of plant physiology new series volume 12 A: Physiological plant ecology I (1st ed., pp. 57–107). Berlin: Springer.

[ece34816-bib-0012] Bliss, L. C. (1962). Adaptations of arctic and alpine plants to environmental conditions. Arctic, 15, 117–144. 10.14430/arctic3564

[ece34816-bib-0013] Bonan, G. B. (2008a). Leaf energy fluxes In Bonan G. B. (Ed.), Ecological climatology (2nd ed., pp. 229–236). Cambridge: Cambridge University Press.

[ece34816-bib-0014] Bonan, G. B. (2008b). Leaf photosynthesis In Bonan G. B. (Ed.), Ecological climatology (2nd ed., pp. 237–252). Cambridge: Cambridge University Press.

[ece34816-bib-0015] Bowman, W. D. , Theodose, T. A. , Schardt, J. C. , & Conant, R. T. (1993). Constraints of nutrient availability on primary production in two alpine tundra communities. Ecology, 74(7), 2085–2097. 10.2307/1940854

[ece34816-bib-0016] Bowman, W. D. (1994). Accumulation and use of nitrogen and phosphorus following fertilization in two alpine tundra communities. Oikos, 70, 261–270. 10.2307/3545637

[ece34816-bib-0017] Bowman, W. D. , Gartner, J. R. , Holland, K. , & Wiedermann, M. (2006). Nitrogen critical loads for alpine vegetation and terrestrial ecosystem response: Are we there yet? Ecological Applications, 16(3), 1183–1193. 10.1890/1051-0761(2006)016[1183:NCLFAV]2.0.CO;2 16827011

[ece34816-bib-0018] Bowman, W. D. , Murgel, J. , Blett, T. , & Porter, E. (2012). Nitrogen critical loads for alpine vegetation and soils in Rocky Mountain National Park. Journal of Environmental Management, 103, 165–171. 10.1016/j.jenvman.2012.03.002 22516810

[ece34816-bib-0019] Bowman, W. D. , Bahn, L. , & Damm, M. (2003). Alpine landscape variation in foliar nitrogen and phosphorus concentrations and the relation to soil nitrogen and phosphorous availability. Arctic, Antarctic, and Alpine Research, 35(2), 144–149.

[ece34816-bib-0020] Bowman, W. D. , & Conant, R. T. (1994). Shoot growth dynamics and photosynthetic response to increased nitrogen availability in the alpine willow *Salix glauca* . Oecologia, 97(1), 93–99. 10.1007/BF00317912 28313593

[ece34816-bib-0021] Bowman, W. D. , & Fisk, M. C. (2001). Primary production In Bowman W. D., & Seastedt T. R. (Eds.), Structure and function of an alpine ecosystem (1st ed., pp. 177–197). New York, NY: Oxford University Press.

[ece34816-bib-0022] Bowman, W. D. , & Steltzer, H. (1998). Positive feedbacks to anthropogenic nitrogen deposition in Rocky Mountain alpine tundra. Ambio, 27(7), 514–517.

[ece34816-bib-0023] Bowman, W. D. , Theodose, T. A. , & Fisk, M. C. (1995). Physiological and production responses of plant growth forms to increases in limiting resources in alpine tundra: Implications for differential community response to environmental change. Oecologia, 101, 217–227. 10.1007/BF00317287 28306794

[ece34816-bib-0024] Castle, S. C. , & Neff, J. C. (2013). What controls plant nutrient use in high elevation ecosystems? Oecologia, 173, 1551–1561. 10.1007/s00442-013-2695-7 23771801

[ece34816-bib-0025] Chapin, F. S. III (1983). Direct and indirect effects of temperature on arctic plants. Polar Biology, 3, 47–52. 10.1007/BF00258285

[ece34816-bib-0026] Chapin, F. S. III (1987). Environmental controls over growth of tundra plants. Ecological Bulletins, 38, 69–76.

[ece34816-bib-0027] Chapin, F. S. III , Autumn, K. , & Pugntairet, F. (1993). Evolution of suites of traits in response to environmental stress. The American Naturalist, 142, S78–S92. 10.1086/285524

[ece34816-bib-0028] Chapin, F. S. III , Matson, P. A. , & Vitousek, P. M. (Eds.) (2012). Plant nutrient use In Principles of terrestrial ecosystem ecology (2nd ed., pp. 229–258). New York, NY: Springer.

[ece34816-bib-0029] Choler, P. (2005). Consistent shifts in alpine plant traits along a mesotopographical gradient. Arctic, Antarctic, and Alpine Research, 37(4), 444–453. 10.1657/1523-0430(2005)037[0444:CSIAPT]2.0.CO;2

[ece34816-bib-0030] Choler, P. , Michalet, R. , & Callaway, R. M. (2001). Facilitation and competition on gradients in alpine plant communities. Ecology, 82(12), 3295–3308. 10.1890/0012-9658(2001)082[3295:FACOGI]2.0.CO;2

[ece34816-bib-0031] Crawford, A. J. , McLachlan, D. H. , Hetherington, A. M. , & Franklin, K. A. (2012). High temperature exposure increases plant cooling capacity. Current Biology, 22(10), R396–R397. 10.1016/j.cub.2012.03.044 22625853

[ece34816-bib-0032] de Bello, F. , Lavorel, S. , Lavergne, S. , Albert, C. H. , Boulangeat, I. , Mazel, F. , & Thuiller, W. (2013). Hierarchical effects of environmental filters on the functional structure of plant communities: A case study in the French Alps. Ecography, 36, 393–402. 10.1111/j.1600-0587.2012.07438.x

[ece34816-bib-0033] de Boer, H. J. , Lammertsma, E. I. , Wagner‐Cremer, F. , Dilcher, D. L. , Wassen, M. J. , & Dekker, S. C. (2011). Climate forcing due to optimization of maximal leaf conductance in subtropical vegetation under rising CO_2_ . Proceedings of the National Academy of Sciences of the United States of America, 108(10), 4041–4046. 10.1073/pnas.1100555108 21330553 PMC3053960

[ece34816-bib-0034] Diaz, H. F. , Bradley, R. S. , & Ning, L. (2014). Climatic changes in mountain regions of the American Cordillera and the tropics: Historical changes and future outlook. Arctic, Antarctic, and Alpine Research, 46(4), 735–743. 10.1657/1938-4246-46.4.735

[ece34816-bib-0035] Diaz, H. F. , & Eischeid, J. K. (2007). Disappearing “alpine tundra” Koppen climatic type in the western United States. Geophysical Research Letters, 34, 1–4.

[ece34816-bib-0036] Diaz, S. , Hodgson, J. G. , Thompson, K. , Cabido, M. , Cornelissen, J. H. C. , Jalili, A. , … Zak, M. R. (2004). The plant traits that drive ecosystems: Evidence from three continents. Journal of Vegetation Science, 15(3), 295–304.

[ece34816-bib-0037] Diaz, S. , Kattge, J. , Cornelissen, J. H. , Wright, I. J. , Lavorel, S. , Dray, S. , … Gorné, L. D. (2016). The global spectrum of plant form and function. Nature, 529, 167–171. 26700811 10.1038/nature16489

[ece34816-bib-0038] Dingman, L. (2014). Physical hydrology (3rd ed.) Long Grove, IL: Waveland Press.

[ece34816-bib-0039] Dreyer, E. , Le Roux, X. , Montpied, P. , Daudet, F. A. , & Masson, F. (2001). Temperature response of leaf photosynthetic capacity in seedlings from seven temperate tree species. Tree Physiology, 21, 223–232. 10.1093/treephys/21.4.223 11276416

[ece34816-bib-0040] Elmendorf , S. C. , Henry , G. H. R. , Hollister , R. D. , Björk , R. G. , Boulanger‐Lapointe , N. , Cooper , E. J. , … Wipf , S. (2012). Plot‐scale evidence of tundra vegetation change and links to recent summer warming. Nature Climate Change, 2, 453–457.

[ece34816-bib-0041] Evans, J. R. (1989). Photosynthesis and nitrogen relationships in leaves of C₃ plants. Oecologia, 78, 9–19. 28311896 10.1007/BF00377192

[ece34816-bib-0042] Fan, Z. , Neff, J. C. , & Wieder, W. R. (2016). Model‐based analysis of environmental controls over ecosystem primary production in an alpine tundra dry meadow. Biogeochemistry, 128, 35–49. 10.1007/s10533-016-0193-9

[ece34816-bib-0043] Farquhar, G. D. , & Sharkey, T. D. (1982). Stomatal conductance and photosynthesis. Annual Review of Plant Physiology, 33, 317–345. 10.1146/annurev.pp.33.060182.001533

[ece34816-bib-0044] Farquhar, G. D. , von Caemmerer, S. , & Berry, J. A. (1980). A biochemical model of photosynthetic CO_2_ assimilation in leaves of C3 species. Planta, 149, 78–90. 10.1007/BF00386231 24306196

[ece34816-bib-0045] Farrer, E. C. , Ashton, I. W. , Spasojevic, M. J. , Fu, S. , Gonzalez, D. J. X. , & Suding, K. N. (2015). Indirect effects of global change accumulate to alter plant diversity but not ecosystem function in alpine tundra. Journal of Ecology, 103, 351–360. 10.1111/1365-2745.12363

[ece34816-bib-0046] Ferrar, P. J. , Slatyer, R. , & Vranjic, J. A. (1989). Photosynthetic temperature acclimation in eucalyptus species from diverse habitats, and a comparison with *Nerium oleander* . Australian Journal of Plant Physiology, 16, 199–217. 10.1071/PP9890199

[ece34816-bib-0047] Field, C. B. , Merino, J. , & Mooney, H. A. (1983). Compromises between water‐use efficiency and nitrogen‐use efficiency in five species of California evergreens. Oecologia, 60, 384–389. 10.1007/BF00376856 28310700

[ece34816-bib-0048] Field, C. B. , & Mooney, H. A. (1986). Photosynthesis‐nitrogen relationship in wild plants In Givnish T. J. (Ed.), On the economy of plant form and function (1st ed., pp.25–55). Cambridge: Cambridge University Press.

[ece34816-bib-0049] Fisk, M. C. (1995). *Nitrogen dynamics in an alpine landscape* . Ph.D. dissertation. University of Colorado, Boulder, CO.

[ece34816-bib-0050] Fisk, M. C. , & Schmidt, S. K. (1995). Nitrogen mineralization and microbial biomass nitrogen dynamics in three alpine tundra communities. Social Science Society of America, 59, 1036–1043. 10.2136/sssaj1995.03615995005900040012x

[ece34816-bib-0051] Fisk, M. C. , Schmidt, S. K. , & Seastedt, T. R. (1998). Topographic patterns of above‐ and belowground production and nitrogen cycling in alpine tundra. Ecology, 79(7), 2253–2266. 10.1890/0012-9658(1998)079[2253:TPOAAB]2.0.CO;2

[ece34816-bib-0052] Flexas, J. , Barbour , M. M. , Brendel , O. , Cabrera , H. M. , Carriquí , M , Díaz‐Espejo , A , … Warren , C. R . (2012). Mesophyll diffusion conductance to CO2: An unappreciated central player in photosynthesis. Plant Science, 193–194, 70–84. 10.1016/j.plantsci.2012.05.009 22794920

[ece34816-bib-0053] Gaastra, P. (1959). Photosynthesis of crop plants as influenced by light, carbon dioxide, temperature, and stomatal diffusion resistance. Mededelingen Van De Landbouwhogeschool Te Wageningen, 59(13), 1–68.

[ece34816-bib-0054] Gasarch, E. I. , & Seastedt, T. R. (2015). Plant community response to nitrogen and phosphorus enrichment varies across an alpine tundra moisture gradient. Plant Ecology & Diversity, 8(5–6), 739–749. 10.1080/17550874.2015.1123317

[ece34816-bib-0055] Givnish, T. J. , & Vermeij, G. J. (1976). Sizes and shapes of liane leaves. American Society of Naturalists, 110(975), 743–778. 10.1086/283101

[ece34816-bib-0056] Greenland, D. (1989). The Climate of Niwot Ridge, Front Range, Colorado, U.S.A. Arctic and Alpine Research, 21(4), 380–391. 10.2307/1551647

[ece34816-bib-0057] Harrison, M. T. , Edwards, E. J. , Farquhar, G. D. , Nicotra, A. B. , & Evans, J. R. (2009). Nitrogen in cell walls of sclerophyllous leaves accounts for little of the variation in photosynthetic nitrogen‐use efficiency. Plant, Cell and Environment, 32, 259–270. 10.1111/j.1365-3040.2008.01918.x 19054350

[ece34816-bib-0058] Hikosaka, K. (1997). Modelling optimal temperature acclimation of the photosynthetic apparatus in C3 plants with respect to nitrogen use. Annals of Botany, 80, 721–730. 10.1006/anbo.1997.0512

[ece34816-bib-0059] Isard, S. A. (1986). Factors influencing soil moisture and plant community distribution on Niwot Ridge, Front Range, Colorado, U.S.A. Arctic and Alpine Research, 18(1), 83–96. 10.2307/1551216

[ece34816-bib-0060] Jaeger, C. H. III , Monson, R. K. , Fisk, M. C. , & Schmidt, S. K. (1999). Seasonal partitioning of nitrogen by plants and soil microorganisms in an alpine ecosystem. Ecology, 80(6), 1883–1891. 10.2307/176666

[ece34816-bib-0061] Kikvidze, Z. , Pugnaire, F. I. , Brooker, R. W. , Choler, P. , Lortie, C. J. , Michalet, R. , & Callaway, R. M. (2005). Linking patterns and processes in alpine plant communities: A global study. Ecology, 86(6), 1395–1400. 10.1890/04-1926

[ece34816-bib-0062] Knowles, J. F. (2015). *Spatio‐temporal patterns of soil respiration and the age of respired carbon from high‐elevation alpine tundra* , Ph.D. dissertation. University of Colorado, Boulder, CO.

[ece34816-bib-0063] Knowles, J. F. , Blanken, P. D. , & Williams, M. W. (2015). Soil respiration variability across a soil moisture and vegetation community gradient within a snow‐scoured alpine meadow. Biogeochemistry, 125, 185–202. 10.1007/s10533-015-0122-3

[ece34816-bib-0064] Korner, C. , & Cochrane, P. (1983). Influence of plant physiognomy on leaf temperature on clear midsummer days in the snowy mountains south‐eastern Australia. Oecologica Plantarum, 4(18), 117–124.

[ece34816-bib-0065] Lambers, H. , Chapin, F. S. III , & Pons, T. L. (Eds.) (2008). Photosynthesis, respiration, and long‐distance transport. In Plant Physiological Ecology (2nd ed., pp. 10–153). New York, NY: Springer.

[ece34816-bib-0066] Larcher, W. (1995). Physiological plant ecology (3rd ed.) Berlin: Springer.

[ece34816-bib-0067] Lenz, K. E. , Host, G. E. , Roskoski, K. , Noormets, A. , Sôber, A. , & Karnosky, D. F. . (2010). Analysis of a Farquhar‐von Caemmerer‐Berry leaf‐level photosynthetic rate model for *Populus tremuloides* in the context of modeling and measurement limitations. Environmental Pollution, 158(4), 1015–1022. 10.1016/j.envpol.2009.08.004 19766365

[ece34816-bib-0068] Leuning, R. (1997). Scaling to a common temperature improves the correlation between the photosynthesis parameters Jmax and Vcmax. Journal of Experimental Botany, 48(307), 345–347.

[ece34816-bib-0069] Lipson, D. A. , Bowman, W. D. , & Monson, R. K. (1996). Luxury uptake and storage of nitrogen in the rhizomatous alpine herb, *Bistorta bistortoides* . Ecology, 77(4), 1277–1285. 10.2307/2265597

[ece34816-bib-0070] Litaor, M. I. , Williams, M. W. , & Seastedt, T. R. (2008). Topographic controls on snow distribution, soil moisture, and species diversity of herbaceous alpine vegetation. Journal of Geophysical Research, 113, 1–10.

[ece34816-bib-0071] Manzoni, S. , Vico, G. , Palmroth, S. , Porporato, A. , & Katul, G. (2013). Optimization of stomatal conductance for maximum carbon gain under dynamic soil moisture. Advances in Water Resources, 62, 90–105. 10.1016/j.advwatres.2013.09.020

[ece34816-bib-0072] McGuire, C. R. , Nufio, C. R. , Bowers, M. D. , & Guralnick, R. P. (2012). Elevation‐dependent temperature trends in the rocky mountain front range: Changes over a 56‐ and 20‐year record. PLoS ONE, 7(9), 1–12. 10.1371/journal.pone.0044370 PMC3435419 22970205

[ece34816-bib-0073] Medlyn, B. E. , Dreyer, E. , Ellsworth, D. , Forstreuter, M. , Harley, P. C. , Kirschbaum, M. U. F. , … Loustau, D. (2002). Temperature response of parameters of a biochemically based model of photosynthesis. II. A review of experimental data. Plant, Cell and Environment, 25, 1167–1179. 10.1046/j.1365-3040.2002.00891.x

[ece34816-bib-0074] Medlyn, B. E. , Loustau, D. , & Delzon, S. (2002). Temperature response of parameters of a biochemically based model of photosynthesis. I. Seasonal changes in mature maritime pine (*Pinus pinaster* Ait.). Plant, Cell and Environment, 25, 1155–1165. 10.1046/j.1365-3040.2002.00890.x

[ece34816-bib-0075] Mooney, H. A. , & Billings, W. D. (1960). The Annual carbohydrate cycle of alpine plants as related to growth. American Journal of Botany, 47(7), 594 10.1002/j.1537-2197.1960.tb14911.x

[ece34816-bib-0076] Natali, S. M. , Schuur, E. A. G. , & Rubin, R. L. (2012). Increased plant productivity in Alaskan tundra as a result of experimental warming of soil and permafrost. Journal of Ecology, 100, 488–498. 10.1111/j.1365-2745.2011.01925.x

[ece34816-bib-0077] Nicotra, A. B. , Cosgrove, M. J. , Cowling, A. , Schlichting, C. D. , & Jones, C. S. (2008). Leaf shape linked to photosynthetic rates and temperature optima in South African *Pelargonium* species. Oecologia, 154(4), 625–635. 10.1007/s00442-007-0865-1 17943318

[ece34816-bib-0078] Niinemets, U. , & Tenhunen, J. D. (1997). A model separating leaf structural and physiological effects on carbon gain along light gradients for the shade‐tolerant species *Acer saccharum* . Plant, Cell and Environment, 20, 845–866. 10.1046/j.1365-3040.1997.d01-133.x

[ece34816-bib-0079] Palmroth, S. , Katul, G. G. , Maier, C. A. , Ward, E. , Manzoni, S. , & Vico, G. (2013). On the complementary relationship between marginal nitrogen and water‐use efficiencies among *Pinus taeda* leaves grown under ambient and CO_2_‐enriched environments. Annals of Botany, 111, 467–477. 10.1093/aob/mcs268 23299995 PMC3579436

[ece34816-bib-0080] Pepin, N. , Bradley, R. S. , Diaz, H. F. , Baraer, M. , Caceres, E. B. , Forsythe, N. , … Yang, D. Q. (2015). Elevation‐dependent warming in mountain regions of the world. Nature Climate Change, 5, 424–430.

[ece34816-bib-0081] Poorter, H. , & Evans, J. R. (1998). Photosynthetic nitrogen‐use efficiency of species that differ inherently in specific leaf area. Oecologia, 116, 26–37. 10.1007/s004420050560 28308535

[ece34816-bib-0082] Radin, J. W. , Lu, Z. , Percy, R. G. , & Zeiger, E. (1994). Genetic variability for stomatal conductance in Pima cotton and its relation to improvements of heat adaptation. Proceedings of the National Academy of Sciences of the United States of America, 91, 7217–7221. 10.1073/pnas.91.15.7217 11607487 PMC44370

[ece34816-bib-0083] Reich, P. B. (2014). The world‐wide “fast–slow” plant economics spectrum: A traits manifesto. Journal of Ecology, 102, 275–301. 10.1111/1365-2745.12211

[ece34816-bib-0084] Reich, P. B. , Ellsworth, D. S. , & Walters, M. B. (1998). Leaf structure (specific leaf area) modulates photosynthesis‐nitrogen relations: Evidence from within and across species and functional groups. Functional Ecology, 12, 948–958. 10.1046/j.1365-2435.1998.00274.x

[ece34816-bib-0085] Rustad, L. E. , Campbell, J. , Marion, G. , Norby, R. , Mitchell, M. , & Hartley, A. , … GCTE‐NEWS (2001). A meta‐analysis of the response of soil respiration, net nitrogen mineralization, and aboveground plant growth to experimental ecosystem warming. Oecologia, 126(4), 543–562. 28547240 10.1007/s004420000544

[ece34816-bib-0086] Salisbury, F. B. , & Spomer, G. G. (1964). Leaf temperatures of alpine plants in the field. Planta, 60, 497–505. 10.1007/BF01894807

[ece34816-bib-0087] Sardinero, S. (2000). Classification and ordination of plant communities along an altitudinal gradient on the Presidential Range, New Hampshire, USA. Plant Ecology, 148, 81–103.

[ece34816-bib-0088] Saxton, K. E. , & Rawls, W. J. (2006). Soil water characteristic estimates by texture and organic matter for hydrologic solutions. Soil Science Society of America Journal, 70(5), 1569–1578. 10.2136/sssaj2005.0117

[ece34816-bib-0089] Scherrer, D. , & Korner, C. (2010). Infra‐red thermometry of alpine landscapes challenges climatic warming projections. Global Change Biology, 16, 2602–2613.

[ece34816-bib-0090] Scherrer, D. , & Korner, C. (2011). Topographically controlled thermal‐habitat differentiation buffers alpine plant diversity against climate warming. Journal of Biogeography, 38, 406–416. 10.1111/j.1365-2699.2010.02407.x

[ece34816-bib-0091] Schlesinger, W. H. , & Bernhardt, E. S. (2013a). The atmosphere In Schlesinger W. H. & Bernhardt E. S. (Ed.), Biogeochemistry: An analysis of global change (3rd ed., pp. 49–91). Oxford: Elsevier.

[ece34816-bib-0092] Schlesinger, W. H. , & Bernhardt, E. S. (2013b). The biosphere: The carbon cycle of terrestrial ecosystems In Schlesinger W. H. & Bernhardt E. S. (Ed.), Biogeochemistry: An analysis of global change (3rd ed., pp. 135–172). Oxford: Elsevier.

[ece34816-bib-0093] Seastedt, T. R. , & Vaccaro, L. (2001). Plant species richness, productivity, and nitrogen and phosphorus limitations across a snowpack gradient in alpine tundra, Colorado, U.S.A. Arctic, Antarctic, and Alpine Research, 33(1), 100–106.

[ece34816-bib-0094] Sievering, H. , Rusch, D. , & Marquez, L. (1996). Nitric acid, particulate nitrate and ammonium in the continental free troposphere: Nitrogen deposition to an alpine tundra ecosystem. Atmospheric Environment, 30(14), 2527–2537. 10.1016/1352-2310(95)00463-7

[ece34816-bib-0095] Singsaas, E. L. , Ort, D. R. , & Delucia, E. H. (2003). Elevated CO_2_ effects on mesophyll conductance and its consequence for interpreting photosynthetic physiology. Plant Cell and Environment, 27, 41–50.

[ece34816-bib-0096] Smith, W. K. (1978). Temperatures of desert plants: Another perspective on the adaptability of leaf size. Science, 201(4356), 614–616. 17794122 10.1126/science.201.4356.614

[ece34816-bib-0097] Soudzilovskaia, N. A. , Onipchenko, V. G. , Cornelissen, J. H. C. , & Aerts, R. (2005). Biomass production, N: P ratio and nutrient limitation in a caucasian alpine tundra plant community. Journal of Vegetation Science, 16, 399–406. 10.1111/j.1654-1103.2005.tb02379.x

[ece34816-bib-0098] Soudzilovskaia, N. A. , Elumeeva, T. G. , Onipchenko, V. G. , Shidakov, I. I. , Salpagarova, F. S. , Khubiev, A. B. , … Cornelissen, J. H. C. (2013). Functional traits predict relationship between plant abundance dynamic and long‐term climate warming. Proceedings of the National Academy of Sciences of the United States of America, 110(45), 18180–18184. 10.1073/pnas.1310700110 24145400 PMC3831435

[ece34816-bib-0099] Spasojevic, M. J. , Bowman, W. D. , Humphries, H. C. , Seastedt, T. R. , & Suding, K. N. (2013). Changes in alpine vegetation over 21 years: Are patterns across a heterogeneous landscape consistent with predictions? Ecosphere, 4(9), 1–18. 10.1890/ES13-00133.1

[ece34816-bib-0100] Spasojevic, M. J. , & Suding, K. N. (2012). Inferring community assembly mechanisms from functional diversity patterns: The importance of multiple assembly processes. Journal of Ecology, 100, 652–661. 10.1111/j.1365-2745.2011.01945.x

[ece34816-bib-0101] Stewart, I. T. (2009). Changes in snowpack and snowmelt runoff for key mountain regions. Hydrological Processes, 23, 78–94.

[ece34816-bib-0102] Stewart, I. T. , Cayan, D. R. , & Dettinger, M. D. (2004). Changes in snowmelt runoff timing in Western North America under a “business as usual” climate change scenario. Climatic Change, 62, 217–232. 10.1023/B:CLIM.0000013702.22656.e8

[ece34816-bib-0103] Stinziano, J. R. , Hüner, N. P. A. , & Way, D. A. (2015). Warming delays autumn declines in photosynthetic capacity in a boreal conifer, Norway Spruce (*Picea abies*). Tree Physiology, 35, 1303–1313. 26543154 10.1093/treephys/tpv118

[ece34816-bib-0104] Suding, K. N. , Lavorel, S. , Chapin, F. S. , Cornelissen, J. H. C. , Díaz, S. , Garnier, E. , … Navas, M.‐L. (2008). Scaling environmental change through the community‐level: A trait‐based response‐and‐effect framework for plants. Global Change Biology, 14, 1125–1140.

[ece34816-bib-0105] Tanaka, K. , Kosugi, Y. , & Nakamura, A. (2002). Impact of leaf physiological characteristics on seasonal variation in CO_2_, latent and sensible heat exchanges over a tree plantation. Agricultural and Forest Meteorology, 114, 103–122. 10.1016/S0168-1923(02)00128-4

[ece34816-bib-0106] Taylor, R. V. , & Seastedt, T. R. (1994). Short‐ and long‐term patterns of soil moisture in alpine tundra. Arctic and Alpine Research, 26(1), 14–20. 10.2307/1551871

[ece34816-bib-0107] Theodose, T. A. , Jaeger, C. H. , Bowman, W. D. , & Schardt, J. C. (1996). Uptake and allocation of 15N in alpine plants: Implications for the importance of competitive ability in predicting community structure in a stressful environment. Oikos, 75, 59–66. 10.2307/3546321

[ece34816-bib-0108] Theodose, T. A. , & Bowman, W. D. (1997). Nutrient availability, plant abundance, and species diversity in two alpine tundra communities. Ecology, 78(6), 1861–1872. 10.1890/0012-9658(1997)078[1861:NAPAAS]2.0.CO;2

[ece34816-bib-0109] Vaughan, D. G. , Comiso, J. C. , Allison, I. , Carrasco, J. , Kaser, G. , Kwok, R. , ... Zhao, L. (2013). *Observations: Cryosphere. Climate Change 2013: The Physical Science Basis. Contribution of Working Group I to the Fifth Assessment Report of the Intergovernmental Panel on Climate Change* .

[ece34816-bib-0110] Vico, G. , Manzoni, S. , Palmroth, S. , Weih, M. , & Katul, G. (2013). A perspective on optimal leaf stomatal conductance under CO2 and light co‐limitations. Agricultural and Forest Meteorology, 182–183, 191–199. 10.1016/j.agrformet.2013.07.005

[ece34816-bib-0111] Vogan, P. J. , & Sage, R. F. (2011). Water‐use efficiency and nitrogen‐use efficiency of C3–C4 intermediate species of *Flaveria Juss*. (Asteraceae). Plant, Cell and Environment, 34, 1415–1430. 10.1111/j.1365-3040.2011.02340.x 21486309

[ece34816-bib-0112] von Caemmerer, S. , & Farquhar, G. D. (1981). Some relationships between the biochemistry of photosynthesis and the gas exchange of leaves. Planta, 153, 376–387. 10.1007/BF00384257 24276943

[ece34816-bib-0113] Walker, A. P. , Beckerman , A. P. , Gu , L. , Kattge , J. , Cernusak, L. A., , T. F. ,… Woodward, F. I. , F. (2014). The relationship of leaf photosynthetic traits—V_cmax_ and J_max_—To leaf nitrogen, leaf phosphorus, and specific leaf area: A meta‐analysis and modeling study. Ecology and Evolution, 4(16), 3218–3235. 25473475 10.1002/ece3.1173 PMC4222209

[ece34816-bib-0114] Walker, M. D. , Walker, D. A. , Welker, J. M. , Arft, A. M. , Bardsley, T. , Brooks, P. D. , … Turner, P. L. (1999). Long‐term experimental manipulation of winter snow regime and summer temperature in arctic and alpine tundra. Hydrological Processes, 13, 2315–2330. 10.1002/(SICI)1099-1085(199910)13:14/15<2315:AID-HYP888>3.0.CO;2-A

[ece34816-bib-0115] Walker, D. A. , Theodose, T. A. , & Webber, P. J. (2001). The vegetation: Hierarchical species environment relationships Structure and function of an alpine ecosystem (1st ed., pp. 99–127). Oxford: Oxford University Press.

[ece34816-bib-0116] Wieder, W. R. , Knowles, J. F. , Blanken, P. D. , Swenson, S. C. , & Suding, K. N. (2017). Ecosystem function in complex mountain terrain: Combining models and long‐term observations to advance process‐based understanding. Journal of Geophysical Research: Biogeosciences, 122, 825–845. 10.1002/2016JG003704

[ece34816-bib-0117] Wipf, S. , Gottfried, M. , & Nagy, L. (2013). Climate change and extreme events—Their impacts on alpine and arctic ecosystem structure and function. Plant Ecology & Diversity, 6(3–4), 303–306. 10.1080/17550874.2013.819533

[ece34816-bib-0118] Wipf, S. , Stoeckli, V. , & Bebi, P. (2009). Winter climate change in alpine tundra: Plant responses to changes in snow depth and snowmelt timing. Climatic Change, 94, 105–121. 10.1007/s10584-009-9546-x

[ece34816-bib-0119] Wohlfahrt, G. , Bahn, M. , Haubner, E. , Horak, I. , Michaeler, W. , Rottmar, K. , … Cernusca, A. (1999). Inter‐specific variation of the biochemical limitation to photosynthesis and related leaf traits of 30 species from mountain grassland ecosystems under different land use. Plant, Cell and Environment, 22, 1281–1296. 10.1046/j.1365-3040.1999.00479.x

[ece34816-bib-0120] Wright, I. J. , Reich, P. B. , Westoby, M. , Ackerly, D. D. , Baruch, Z. , Bongers, F. , … Villar, R. (2004). The worldwide leaf economics spectrum. Nature, 428, 821–827. 10.1038/nature02403 15103368

[ece34816-bib-0121] Zhao, Y. , Ali, A. , & Yan, E. (2016). The plant economics spectrum is structured by leaf habits and growth forms across subtropical species. Tree Physiology, 37, 173–185. 10.1093/treephys/tpw098 28399260

