# Supplementary material for: Leaf temperatures mediate alpine plant communities’ response to a simulated extended summer
Source: Ecol Evol. 2018 Dec 28;9(3):1227–43. doi: 10.1002/ece3.4816 (PMC6374730; doi:10.1002/ece3.4816)
Supplement: Supplementary file 1 [file ECE3-9-1227-s001.docx]

SUPPLEMENTARY MATERIALS

**Supplementary Equations**

$\Gamma^{*}$ is the CO_2_ compensation point during leaf photosynthesis (Farquhar et al. 1980; Equation S1). See Tables 1-3 in paper for parameter definitions.

| $\Gamma^{*}=\left( \frac{K_{c25}\left[ O_{2} \right]}{2K_{o25}} \right)t_{oc}$ | (S1) |
| --- | --- |

The rubisco-limited rate of assimilation is determined by the rate of carboxylation of RuBP by rubisco in the Calvin Cycle (*V_cmax_*). *V_cmax_* is a function of the nitrogen content in rubisco (*nr*), the fraction of leaf nitrogen allocated to rubisco (*flnr*), the specific activity of rubisco (*ra*), and the leaf nitrogen content (*na*) (Niinemets & Tenhunen, 1997; Equation S2).

| $V_{cmax}=nr\cdot flnr\cdot ra\cdot na$ | (S2) |
| --- | --- |

The RuBP-limited rate of assimilation is determined by the rate of electron transport during the light reaction (*J*). The model variable *J* is a function of the thylakoid chlorophyll content (*chl*) and photosynthetically active radiation (*PAR*) (Björkman, 1981; Evans, 1996; Harley & Tenhunen, 1991; Niinemets & Tenhunen, 1997; Equation S3, S4). Additionally, *J* is a function of the maximum rate of electron transport (*J_max_*), which is proportional to *V_cmax_* (Leuning 1997; Wullschleger 1993; Equation S5) .

|  | $J=\frac{(qalpha)(PAR)}{\sqrt{1+\frac{qalpha^{2}PAR^{2}}{J_{max}^{2}}}}$ | (S3) |
| --- | --- | --- |
|  | $qalpha=\frac{(chl/1000)}{(chl/1000)+0.076}(qeff)$ | (S4) |
|  | $J_{max}={j_{m}\cdot V}_{cmax}$ | (S5) |

The parameters $\Gamma^{*}$, *K_c_* , and *K_o_* are a function of leaf temperature (*t_l_*) according to the Arrhenius Equation (Medlyn, Dreyer, et al., 2002; Equation S6). In addition, *V_cmax_*, the maximum rate of carboxylation, and *J*, the rate of electron transport, are a function of leaf temperature according to the Boltzmann Distribution (Medlyn, Loustau, & Delzon, 2002; Equation S7).

|  | $f\left( t_{l} \right)=X_{25}\text{exp}\left( \frac{e_{X}\left( t_{l}-298 \right)}{298Rt_{l}} \right)$ | (S6) |
| --- | --- | --- |
|  | $f(t_{l})=X(t_{opt})\frac{h_{d}\text{exp}\left( \frac{e_{X}\left( t_{l}-t_{opt} \right)}{Rt_{l}t_{opt}} \right)}{h_{d}-e_{X}\left( 1-\text{exp}\left( \frac{h_{d}\left( t_{l}-t_{opt} \right)}{Rt_{l}t_{opt}} \right) \right)}$ | (S7) |

The rate of assimilation depends on the concentration of CO_2_ in the chloroplast (*C_c_*). The model variable *C_c_* is determined by calculating (1) the boundary layer conductance (*g_b_*) as a function of leaf diameter (Equation S8) (2) the stomatal conductance (*g_s_*) as a function of *A*, relative humidity (rh), and the concentration of CO_2_ at the leaf surface (*C_s_*) (Equation S9), and (3) the mesophyll conductance (*g_m_*) (Collatz et al. 1991; Ball et al. 1987; Givnish & Vermeij 1976; Bonan 2008a). We set mesophyll conductance equal to stomatal conductance in the model (Lambers et al. 2008; Equation S9). Additionally, *g_b_*, *g_s_*, and *g_m_* can be written in terms of *A*, the atmospheric concentration of CO_2_ (*C_a_*), *C_s_*, the mesophyll concentration of CO_2_ (*C_i_*), and *C_c_* using Ohm’s Law (Lambers et al. 2008; Bonan 2008a; Bonan 2008b; Equations S10-S12).

|  | $g_{b}=\frac{D_{b}\cdot b}{200\sqrt{dia/u}}$ | (S8) |
| --- | --- | --- |
|  | $g_{s}=g_{m}=\frac{a\cdot m\cdot A\cdot rh+C_{s}\cdot a\cdot g_{0}}{C_{s}}$ | (S9) |
|  | $g_{b}=\frac{A}{C_{a}-C_{s}}$ | (S10) |
|  | $g_{s}=\frac{A}{C_{s}-C_{i}}$ | (S11) |
|  | $g_{m}=\frac{A}{C_{i}-C_{c}}$ | (S12) |

To solve for *A* and *C_c_*, we expanded Baldocchi (1994)’s approach to include a term for mesophyll conductance, which is no longer considered infinite (Lambers et al. 2008; Singsaas et al. 2003). Equations 5-8 are combined to generate a polynomial where *C_c_* is a function of *A* (Equations S13-S18).

|  | $C_{c}=\frac{A^{2}(X1)+A(X2)+(X3)}{A(X4)+(X5)}$ | (S13) |
| --- | --- | --- |

A and C_c_ can then be solved for with a system of equations by combining Equation 1, simplified to $A=\frac{a_{1}(C_{c}-\Gamma^{*})}{C_{c}+a_{2}}$ where a_1_ and a_2_ represent grouped kinetic parameters, with Equation S13 (Equation 4).

We use the vapor pressure deficit to calculate the transpiration term in WUE (Equation 19). Vapor pressure deficit (*vpd*) refers to the difference in vapor pressure between the leaf (*e_l_^*^*) and the air (*e_a_^*^*) (Dingman, 2014; Equation S24). The *e_l_^*^* is a function of leaf temperature (*t_l_*) and *e_a_^*^* is a function of surface air temperature (*t*) and relative humidity (*rh*) (Dingman, 2014; Equation S25-S27).

|  | | $vpd=e_{l}^{*}-e_{a}$ | (S14) |
| --- | --- | --- | --- |
|  | | $e_{l}^{*}=611\cdot\text{exp}\left( \frac{17.27\cdot t_{l}}{t_{l}+237.3} \right)$ | (S15) |
|  | | $e_{a}^{*}=611\cdot\text{exp}\left( \frac{17.27\cdot t}{t+237.3} \right)$ | (S16) |
|  | $e_{a}=rh\cdot e_{a}^{*}$ | | (S17) |

The available volumetric soil moisture content (*vwc-fc*) and transpiration (*T*) and are converted into L/m^2^ (*vwc_a_* and *T_a_*, respectively; Equation S28, S29).

|  | $vwc_{a}=1000\left( vwc-fc \right)z$ | (S18) |
| --- | --- | --- |
|  | $T_{a}={T\cdot10}^{-6}\cdot\frac{1}{M_{w}}\cdot43200$ | (S19) |

**Supplementary Figures**

Figure S1: The difference between leaf and surface temperature as a function of leaf height above the ground in a high elevation ecosystem. y=-0.4366x+18.3381; r^2^=0.2209. Plotted values include all elevations and plant types except for trees (Korner & Cochrane 1983).

| 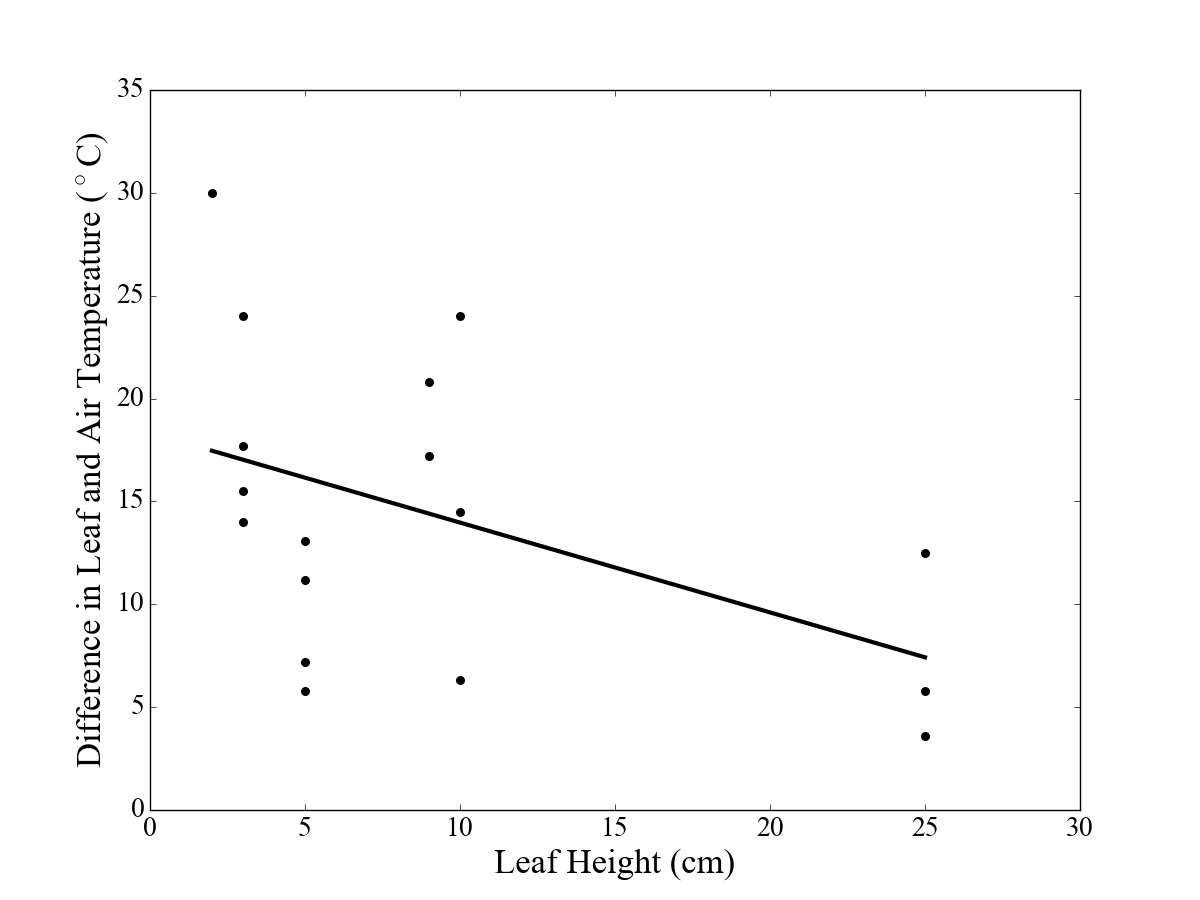 |
| --- |

Figure S2: (A) Simulated maximum assimilation rates in dry and wet meadow species during the height of summer (mid-July through mid-August). The boxplot shows the mean (point), median (line), 50^th^ and 99^th^ percentile range (box and whiskers, respectively) of assimilation values when the model is run with parameter uncertainty. (B) Average growth rates calculated using Niwot Ridge LTER snow cover, temperature, and peak biomass datasets for the saddle grid (http://niwot.colorado.edu). Mean and standard deviations are calculated from growth rates in various representative plant communities for the years 2011-2014. Dry meadow: N=16 plant communities*4 years; Moist meadow: N=22 plant communities*4 years; Wet meadow: N=5 plant communities*4 years; P<0.05.

| 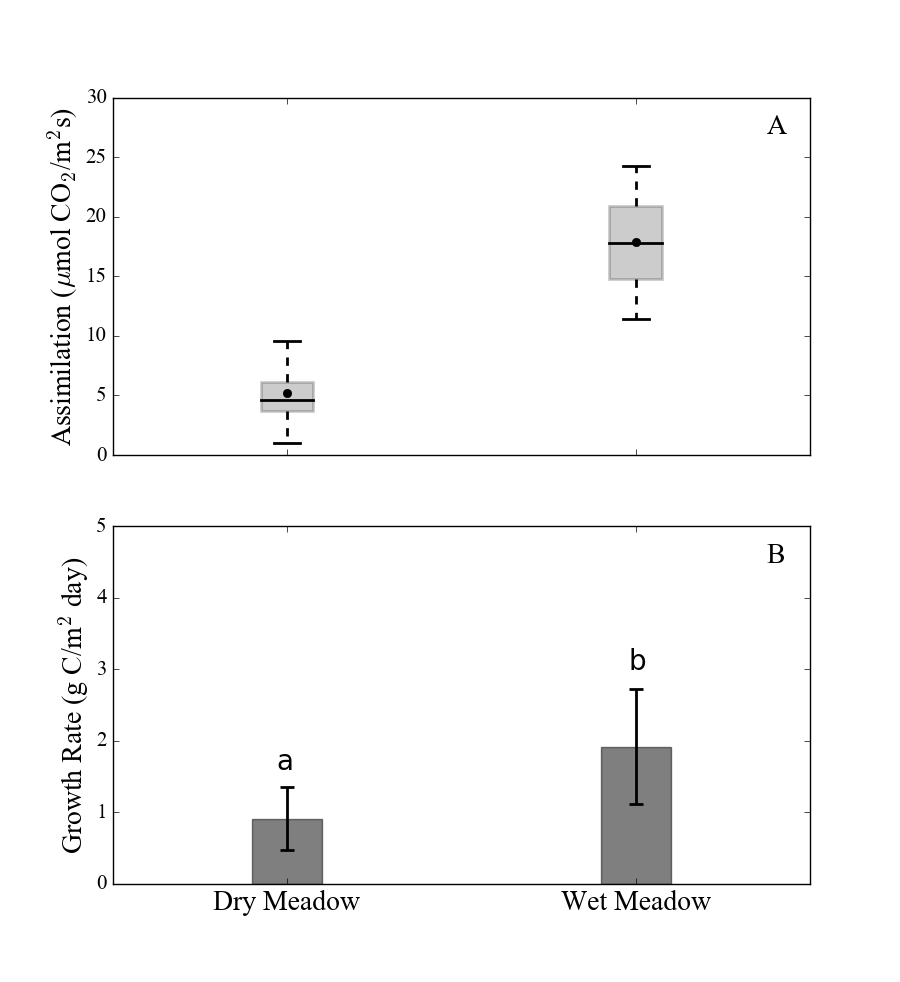 |
| --- |

Figure S3: (A) Simulated NUE in dry and wet meadow species during the height of summer (mid-July through mid-August). The boxplot shows the mean (point), median (line), 50^th^ and 99^th^ percentile range (box and whiskers, respectively) of NUE values when the model is run with parameter uncertainty. (B) Empirical NUE measured as the ratio of senescent biomass to senescent nitrogen (integrated NUE). Measurements taken at Niwot Ridge. N=3; P<0.05 (Fisk et al. 1998).


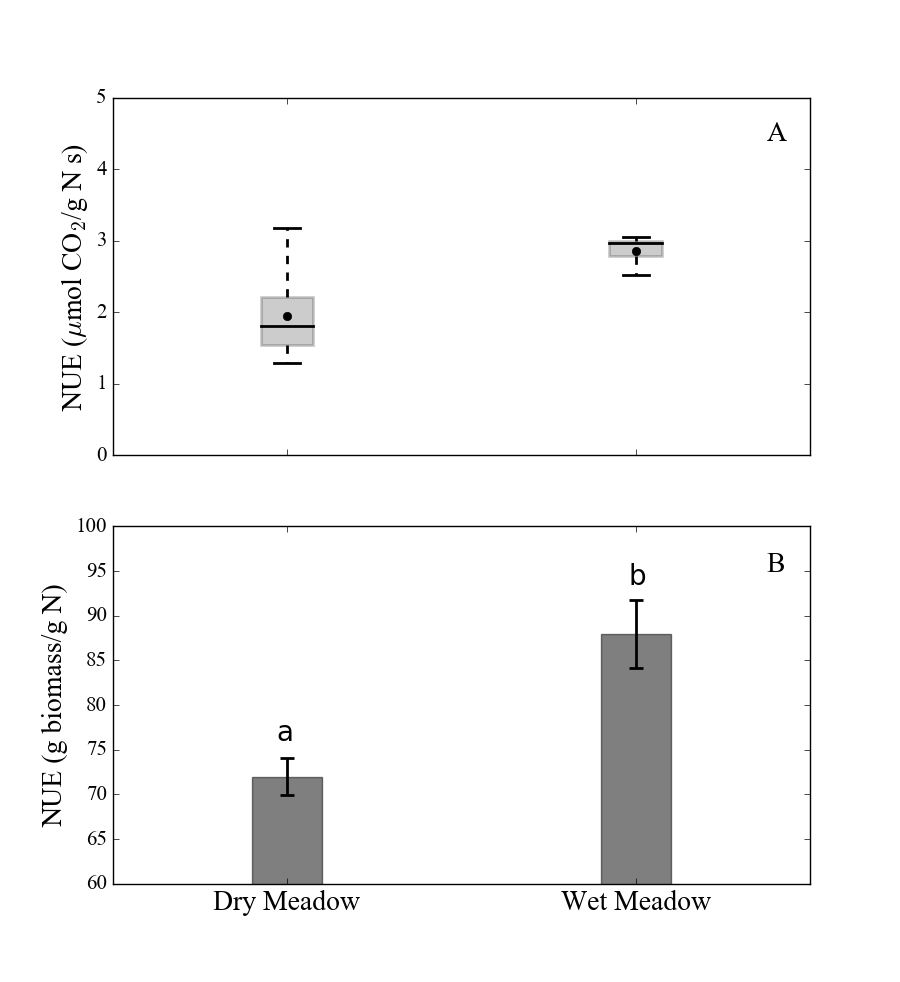


Figure S4: (A) Simulated WUE in dry and moist meadow species during the height of summer (mid-July through mid-August). The boxplot shows the mean (point), median (line), 50^th^ and 99^th^ percentile range (box and whiskers, respectively) of WUE values when the model is run with parameter uncertainty. Model is run with varying leaf temperature and constant leaf temperature. (B) Empirical WUE observed in the dry and moist meadow plant communities at Niwot Ridge. Leaf temperature is constant across plant communities. N=3-4; P<0.05 (Bowman et al. 1995).

|  |
| --- |

Figure S5: Simulated assimilation and stomatal conductance in Niwot Ridge dry and wet meadow species at the height of the growing season (mid-July through mid-August). Multiple simulations (points) account for parameter uncertainty.

| 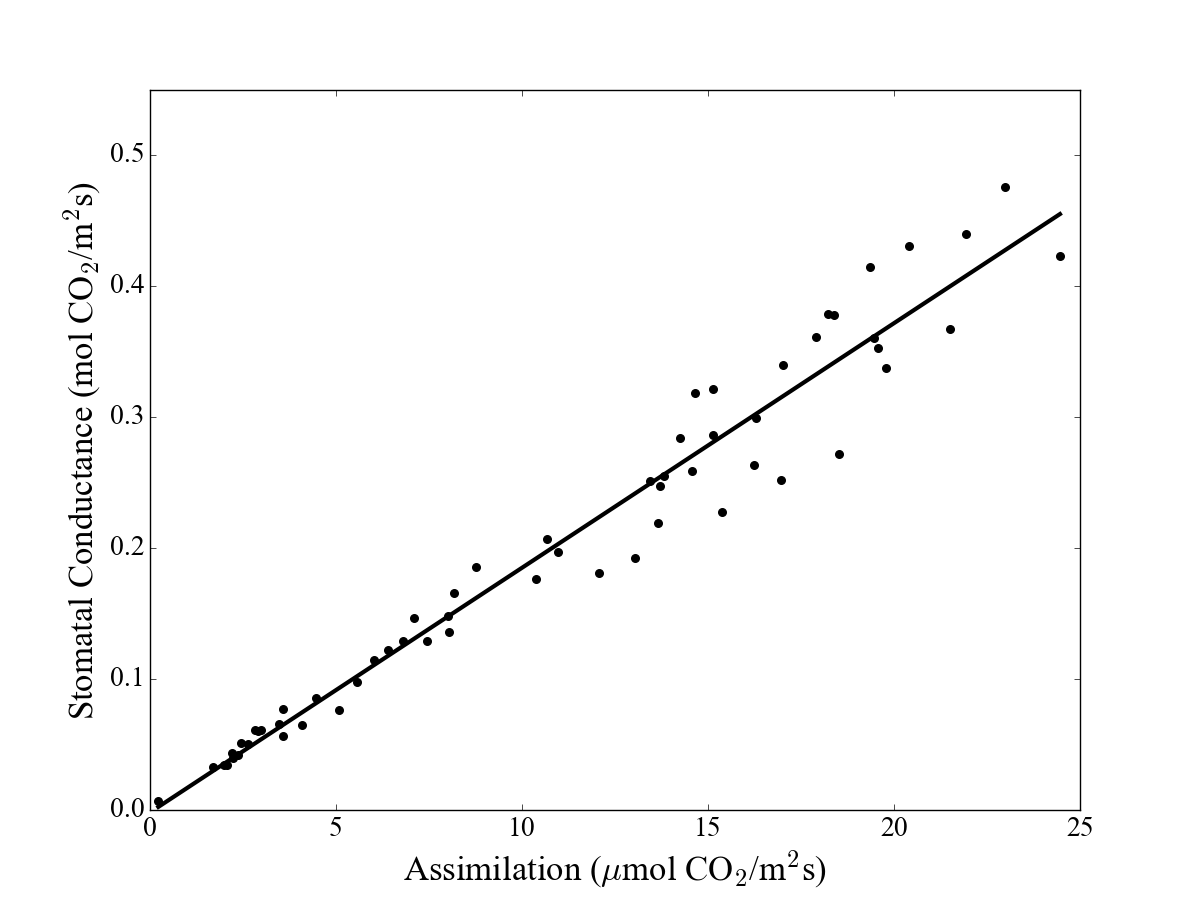 |
| --- |

Figure S6: (A) Observed WUE as a function of vapor pressure deficit (VPD) in two common alpine tundra plants (Deschampsia caespitosa and Geum rossii) at Niwot Ridge (Johnson & Caldwell 1975). (B) Simulated vapor pressure deficit (VPD) and WUE in Niwot Ridge dry and wet meadows at the height of the growing season (mid-July through mid-August). Multiple simulations (points) account for parameter uncertainty.

| 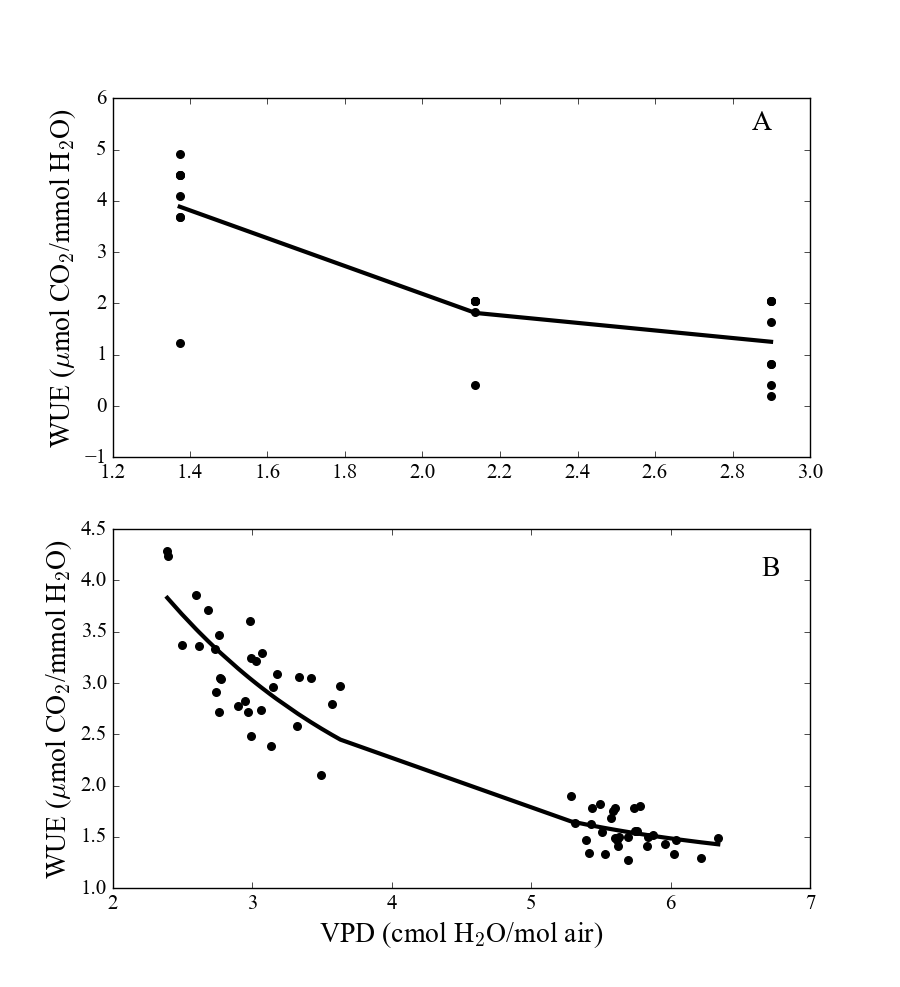 |
| --- |

**Supplementary Tables**

Table S1: Snowmelt timing in an average and extended summer year for dry and wet meadow environments. The start of the average year growing season in the dry and wet meadows was estimated as the first day without snow cover averaged from 1993-2014 at Niwot Ridge (http://niwot.colorado.edu). The final date of the growing season was estimated from soil respiration data measured from 2011-2013 at Niwot Ridge (John Knowles, unpublished data).

|  | Dry Meadow Snowmelt Date | Wet Meadow Snowmelt Date | End of the Growing Season Date |
| --- | --- | --- | --- |
| Average Year | 4/20 | 5/24 | 8/27 |
| 2012 Extended Summer Year | 3/21 | 4/24 | 8/27 |

Table S2: Average abiotic and leaf trait parameters that are specific to the moist meadow species during the height of a typical growing season at Niwot Ridge (July 15-August 15). Mean values given. Standard deviations in parentheses. All parameters are adjusted for the influence of elevation.

| Acronym | Definition | Units | Moist Meadow Species Values | References |
| --- | --- | --- | --- | --- |
| vwc | Midsummer Volumetric  Soil Moisture Content | m^3^ m^-3^ | 0.15 | http://niwot.colorado.edu (2013 and 2014 average) |
| t | Midsummer Maximum Surface Temperature | $℃$ | 15 | Scherrer & Körner (2011); http://niwot.colorado.edu (2013 and 2014 average) |
| z | Soil Depth | m | 0.2 | http://niwot.colorado.edu |
| chl | Leaf Chlorophyll Content | μmol Chl m ^-2^ | 465 (54) | Spasojevic et al. (2013) |
| ht | Leaf Height | cm | 19.2 (5.7) | Spasojevic et al. (2013) |
| dia | Leaf Diameter | cm | 2.6 (1.3) | Spasojevic et al. (2013) |
| na | Leaf Nitrogen Content | g N m^-2^ | 5.0 (1.4) | Fisk (1995) |

**Supplementary Text**

Discussion of Model Validation

Modeled rates of assimilation, stomatal conductance, and transpiration across dry and wet meadow plant communities are similar to empirical measurements calculated for alpine regions. Although the modeled instantaneous NUE is different than the observed instantaneous NUE, the observed NUE has only one alpine plant species as a data point (Bowman et al. 1995). Like Fisk, Schmidt, & Seastedt (1998), Bowman (1994) also measured the integrated NUE across plant communities; however, he observed that the wet meadow plant species have lower NUE (85 g biomass g N^-1^) than the dry meadow (105 g biomass g N^-1^). The integrated NUE is a rough substitute for instantaneous NUE because it includes a wide variety of physiological processes outside of the leaf, such as the partitioning of nutrients between resorption and decomposition pathways (Aerts & Chapin III 2000). Because of these conflicting results and lack of data, we cannot verify trends in instantaneous NUE across dry and wet meadow plant communities. The observed WUE differs from the modeled WUE because Bowman et al. (1995)’s observations were made using a Licor LI-6400 gas chamber, which kept leaf temperature at a constant 20°C. The transpiration term in the modeled WUE is largely a function of the vapor pressure deficit (VPD) which varies with the leaf temperature; this may be where the model and field data diverge. When the model was run a second time with a constant leaf temperature in dry and moist meadow species, the modeled WUE for the plant communities is at a similar constant value (1.8 µmol CO_2_/mmol H_2_O) as the empirical data (Supplementary Materials: Figure S4). Moreover, Johnson & Caldwell (1975) observed that when VPD increased, the measured instantaneous WUE declined at a decreasing rate. Model simulations show a similar trend (Supplementary Materials: Figure S6).

REFERENCES

Aerts, R. & Chapin III, F.S., 2000. The Mineral Nutrition of Wild Plants Revisited: A Re-evaluation of Processes and Patterns. *Advances in Ecological Research*, 30, pp.1–67.

Ball, J.T., Woodrow, I.E. & Berry, J.A., 1987. A Model Predicting Stomatal Conductance and its Contribution to the Control of Photosynthesis under Different Environmental Conditions. *Progress in Photosynthesis Research*, 4(5), pp.221–224.

Bjorkman, O., 1981. Responses to Different Quantum Flux Densities. In *Physiological Plant Ecology I*. pp. 57–107.

Bonan, G.B., 2008a. Leaf Energy Fluxes. In *Ecological Climatology*. Cambridge University Press, pp. 229–236.

Bonan, G.B., 2008b. Leaf Photosynthesis. In *Ecological Climatology*. Cambridge University Press, pp. 237–252.

Bowman, W.D., 1994. Accumulation and Use of Nitrogen and Phosphorus Following Fertilization in Two Alpine Tundra Communities. *Oikos*, 70, pp.261–270.

Bowman, W.D., Theodose, T.A. & Fisk, M.C., 1995. Physiological and Production Responses of Plant Growth Forms to Increases in Limiting Resources in Alpine Tundra: Implications for Differential Community Response to Environmental Change. *Oecologia*, 101, pp.217–227.

Collatz, G.J. et al., 1991. Physiological and Environmental Regulation of Stomatal Conductance, Photosynthesis and Transpiration: A Model that Includes a Laminar Boundary Layer. *Agricultural and Forest Meteorology*, 54, pp.107–136.

Dingman, L., 2014. *Physical Hydrology* 3rd ed., Waveland Press.

Evans, J.R., 1996. Developmental Constrains on Photosynthesis: Effects of Light and Nutrition. *Photosynthesis and the Environment*, pp.281–304.

Farquhar, G.D., von Caemmerer, S. & Berry, J.A., 1980. A Biochemical Model of Photosynthetic CO2 Assimilation in Leaves of C3 Species. *Planta*, 149, pp.78–90.

Fisk, M.C., 1995. *Nitrogen Dynamics in an Alpine Landscape. Ph.D. Dissertation*. University of Colorado, Boulder.

Fisk, M.C., Schmidt, S.K. & Seastedt, T.R., 1998. Topographic Patterns of Above- and Belowground Production and Nitrogen Cycling in Alpine Tundra. *Ecology*, 79(7), pp.2253–2266.

Givnish, T.J. & Vermeij, G.J., 1976. Sizes and Shapes of Liane Leaves. *American Society of Naturalists*, 110(975), pp.743–778.

Harley, P.C. & Tenhunen, J.D., 1991. Modeling the Photosynthetic Response of C3 Leaves to Environmental Factors. *Modeling Crop Photosynthesis from Biochemistry to Canopy*, pp.17–39.

Johnson, D.A. & Caldwell, M.M., 1975. Gas Exchange of Four Arctic and Alpine Tundra Plant Species in Relation to Atmospheric and Soil Moisture Stress. *Oecologia*, 21, pp.93–108.

Korner, C. & Cochrane, P., 1983. Influence of Plant Physiognomy on Leaf Temperature on Clear Midsummer Days in the Snowy Mountains South-Eastern Australia. *OEcologica Plantarum*, 4(18), pp.117–124.

Lambers, H., Chapin III, F.S. & Pons, T.L., 2008. Photosynthesis, Respiration, and Long-Distance Transport. In *Plant Physiological Ecology*. pp. 11–162.

Leuning, R., 1997. Scaling to a Common Temperature Improves the Correlation Between the Photosynthesis Parameters Jmax and Vcmax. *Journal of Experimental Botany*, 48(307), pp.345–347.

Medlyn, B.E., Dreyer, E., et al., 2002. Temperature Response of Parameters of a Biochemically Based Model of Photosynthesis. II. A Review of Experimental Data. *Plant, Cell and Environment*, 25, pp.1167–1179.

Medlyn, B.E., Loustau, D. & Delzon, S., 2002. Temperature Response of Parameters of a Biochemically Based Model of Photosynthesis. I. Seasonal Changes in Mature Maritime Pine (Pinus pinaster Ait.). *Plant, Cell and Environment*, 25, pp.1155–1165.

Niinemets, U. & Tenhunen, J.D., 1997. A Model Separating Leaf Structural and Physiological Effects on Carbon Gain Along Light Gradients for the Shade-Tolerant Species Acer saccharum. *Plant, Cell and Environment*, 20, pp.845–866.

Scherrer, D. & Korner, C., 2011. Topographically Controlled Thermal-Habitat Differentiation Buffers Alpine Plant Diversity Against Climate Warming. *Journal of Biogeography*, 38, pp.406–416.

Singsaas, E.L., Ort, D.R. & Delucia, E.H., 2003. Elevated CO2 Effects on Mesophyll Conductance and its Consequence for Interpreting Photosynthetic Physiology. *Plant Cell and Environment*, 27, pp.41–50.

Spasojevic, M.J. et al., 2013. Changes in Alpine Vegetation Over 21 Years: Are Patterns Across a Heterogeneous Landscape Consistent with Predictions? *Ecosphere*, 4(9), pp.1–18.

Wullschleger, S.D., 1993. Biochemical Limitations to Carbon Assimilation in C3 Plants-A Retrospective Analysis of the A/Ci Curves from 109 Species. *Journal of Experimental Botany*, 44(262), pp.907–920.
